# Systematic review: the relationship between sleep spindle activity with cognitive functions, positive and negative symptoms in psychosis

**DOI:** 10.1016/j.sleepx.2020.100025

**Published:** 2020-08-29

**Authors:** Chi Hung Au, Christopher-James Harvey

**Affiliations:** a Queen Mary Hospital, Hong Kong; b Imperial College London, UK

**Keywords:** Sleep spindle, Psychosis, Cognitive function, Positive symptom, Negative symptom

## Abstract

**Background:**

Sleep disturbances are associated with worse cognitive and psychotic symptoms in individuals with schizophrenia. Growing literature reveals sleep spindle deficits in schizophrenia may be an endophenotype reflecting a dysfunctional thalamo-thalamic reticular nucleus-cortical circuit. Since thalamic functions link to cognitive, positive and negative symptoms, it is possible that sleep spindle activity is associated with these symptoms. The primary objectives of this systematic review were to assess the associations of sleep spindle activity in psychotic patients with 1) cognitive functions; and 2) positive and negative symptom severity. A secondary objective was to examine which spindle parameter would be the most consistent parameter correlating with cognitive functions, and positive and negative symptoms.

**Method:**

Observational studies reporting an association between sleep spindle activity and cognitive functions, positive and negative symptoms in patients with psychotic disorders were considered eligible. We developed a comprehensive electronic search strategy to identify peer-reviewed studies in Pubmed, Embase, PsycINFO and CINAHL covering all dates up to the search date in May 2020 with no language restriction. The references of published articles were hand-searched for additional materials. The authors of published articles were contacted for newer or unpublished data. Risk of bias was assessed by Appraisal of Cross-sectional Studies (AXIS).

**Results:**

A total 11 cross-sectional studies (n = 255) with low-to-moderate quality, were selected for the systematic review. 8 of them addressed the association between sleep spindle activity and cognitive functions (n = 193), of which 6 studies reported positive correlations (r only reported in 4 studies, from 0.45 to 0.75). Out of multiple cognitive domains, we have only found attention/cognitive processing speed to have a more consistent positive association with sleep spindle activity. On the other hand, 8 studies investigated the relationship between sleep spindle and positive/negative symptom severity (n = 190), but findings were inconsistent. Spindle density is the most consistent parameter correlating with cognitive functions, while the best spindle parameter for correlating with positive and negative symptom severity cannot be identified due to mixed results.

**Discussion:**

This systematic review confirms the linkage between sleep spindle activity and cognitive functions. However, included studies had small sample sizes, with high risks of sampling and response bias. Moreover, confounders were often not controlled. The heterogeneous report of spindle parameters and use of cognitive assessment tools rendered meta-analysis infeasible. It is necessary to examine the longitudinal change of sleep spindle activity with the course of illness, as well as the effect of sleep spindle enhancing agents on cognitive function.

## Highlights

• Spindle activity positively correlates with cognitive functions (especially attention/cognitive processing speed) in individuals with psychotic disorders.
• Spindle density is the most consistent spindle parameter correlating with cognitive functions.
• The relationship between spindle activity and positive/negative symptoms is inconclusive.
• Future studies should address the longitudinal change of spindle activity.
• The effect of spindle enhancing agents in individuals with psychotic disorders should be studied.

## 1 Background

### 1.1 What are sleep spindles?

Sleep spindles are waxing and waning oscillatory waveforms with a frequency between 9 and 16 Hz that appear during the non-rapid eye movement sleep [1]. Apart from the more well-characterised slow oscillations, growing evidence supports that sleep spindles have a central role in enhancing memory consolidation. After sleep spindles are generated in the thalamic reticular nucleus (TRN), they are relayed to the cortex through the thalamocortical circuit and increase glutamate receptors expression so that the neocortex become a suitable environment for long-term potentiation [2]. Sleep spindles are synchronised with hippocampal sharp-wave ripples by slow oscillations so that the temporal sequence replay in the hippocampus coincides with the readiness for encoding in the neocortex, thereby enhancing the hippocampal-neocortex transfer of information [3,4]. Overall, sleep spindles are neural oscillations which facilitate memory consolidation through coordination with hippocampal ripples and slow oscillations.

### 1.2 Sleep spindles and schizophrenia

Sleep disturbances have been well described in individuals with schizophrenia [5], and are associated with worse psychotic symptoms and cognitive impairments [6,7]. Sleep spindle deficit is one of the most consistent sleep disturbances in schizophrenia. It is present in both individuals with early-onset [8] and chronic schizophrenia [9,10]. Intriguingly, sleep spindle activity is diminished in first-degree relatives of schizophrenia patients indicating a potential genetic component [11]. Therefore, sleep spindles may be involved in the pathophysiology of schizophrenia.

### 1.3 Sleep spindle deficits represent abnormal thalamocortical circuit function in schizophrenia

Sleep spindle deficit in schizophrenia is linked to a dysfunctional thalamocortical circuit. A meta-analysis of magnetic resonance imaging (MRI) studies reveals that the thalamus is smaller in both individuals with first-episode psychosis (FEP) and chronic schizophrenia relative to healthy controls [12]. An MRI-high density electroencephalography (hd-EEG) study further shows that a smaller thalamus is associated with decreased sleep spindle activity in individuals with schizophrenia [13]. In addition, their connectivity of thalamus with the prefrontal cortex is reduced [14], while the connectivity with the sensorimotor cortical areas is increased [15]. Bengi Baran et al. [16] further confirmed that the increased connectivity between the thalamus and sensorimotor cortex is linked to a decreased spindle activity. Thus, the reduced sleep spindle activity in schizophrenia likely reflects the underlying dysfunctional thalamocortical circuit.

### 1.4 Sleep spindle deficits could be an endophenotype of schizophrenia

Sleep spindle deficit may be an endophenotype of schizophrenia because experimental deactivation of two schizophrenia-related genes, CACNA1 and mGluR5, are associated with diminished sleep spindle activities. CACNA1 is responsible for encoding CaV3.3 calcium channel largely in the TRN and is shown to be associated with schizophrenia [17]. The knockout of CACNA1i results in weakened sleep spindle activity in mice [18]. Similarly, mGluR5 encodes the type 5 metabotropic glutamate receptor. This is implicated in NMDA receptor activity, which is dysregulated in schizophrenia. By knocking out mGluR5, the mice exhibited a diminished sleep spindle activity [19]. Hence, the sharing of common genetic factors by sleep spindle and schizophrenia supports that sleep spindle deficit could be a trait in schizophrenia.

### 1.5 A possible link between sleep spindle activity and cognitive functions in schizophrenia

Multiple cognitive functions are impaired in individuals with schizophrenia and these could be associated with sleep spindle deficits. They exhibit impaired executive function, working memory, verbal memory, visual memory, attention, speed of processing, and social cognition [20,21]. Intelligence has been shown to deteriorate as the illness progresses [21]. Sleep spindle activity is enhanced with verbal and procedural learning [[22], [23], [24]], and associated with full-scale and fluid intelligence [25,26]. Therefore, it is meaningful to examine the association between sleep spindle activity and cognitive function in individuals with schizophrenia. However, cognitive function is an umbrella term and which specific cognitive domain being related to sleep spindle activity remains unclear. Thus, apart from cognitive function as an overall study area, specific cognitive domains should also be evaluated.

### 1.6 A possible link between sleep spindle activity and positive/negative symptoms in schizophrenia

Both positive and negative symptoms of schizophrenia are linked to the thalamus and may, therefore, be related to sleep spindle activity. In individuals with FEP, the volume of thalamus is reduced relative to healthy controls, and this is positively associated with the severity of delusions and hallucinations [27,28]. Furthermore, positive and negative symptoms are related to specific abnormal cerebral perfusion patterns over various structures including the thalamus, as shown by a single-photon emission computed tomography study [29]. Before any antipsychotic treatments, delusions and hallucinations are strongly associated with hypoperfusion of left thalamic, cingulate, and frontal cortices, whereas negative symptoms are not associated with abnormal thalamic perfusion. On the contrary, after antipsychotic treatments, only negative but not positive symptoms become associated with hypoperfusion of thalamic cortices. Individuals with schizophrenia also have an overconnectivity between thalamus and sensorimotor cortex, which is positively correlated with the overall symptom severity [30]. Given that both sleep spindle activity and positive/negative symptoms are related to the thalamus, it is conceivable to postulate an association between them.

### 1.7 Which spindle parameter has the most consistent association with cognitive and positive/negative symptoms?

Various spindle parameters have been used in different studies, including spindle number, density (total spindle number divided by a given period, usually stage 2 (N2) sleep), duration, amplitude, frequency, integrated spindle activity (ISA, integrating the absolute amplitude values of each spindle over time), and sigma power. However, most studies only examined some of the parameters and they do not necessarily agree with each other. For example, Manoach et al. [11] found that cognitive function were positively correlated with both spindle density and amplitude, while positive symptoms were positively associated with spindle amplitude only. Therefore, identification of the most consistent corresponding spindle parameter with cognitive function and positive/negative symptoms is necessary to guide future research.

Although narrative reviews by Manoach et al. [31] and Ferrarelli and Tononi [32] suggested relationships between sleep spindle activity, cognitive functions and positive symptoms in the population with psychosis, they did not examine which spindle parameter(s) has the most consistent association with cognitive functions and positive/negative symptoms. This systematic review aims to address this specific gap in the literature.

### 1.8 Objectives

Primary objectives were to examine associations between 1) sleep spindle activity and cognitive functions; and 2) sleep spindle activity and positive/negative symptoms, in individuals with psychotic disorders.

The secondary objective was to examine the most consistent corresponding spindle parameter with cognitive functions and positive/negative symptoms.

## 2 Methods

### 2.1 Evidence acquisition

This research synthesis was done in accordance with the PRISMA statement for the reporting of systematic reviews and meta-analyses [33].

### 2.2 Eligibility criteria

Inclusion criteria:

Patient: Adults with psychotic disorders (age≥18).
Intervention: None.
Comparison: Optional.
Outcome: Associations between sleep spindle activity and cognitive function, positive or negative symptoms.
Type of studies: Observational studies.

Exclusion criteria: 1) Psychotic disorders were due to a general medical condition or substance use; 2) Study subjects had comorbid diagnoses such as major depressive disorder and personality disorders; or 3) Studies did not report how the sleep spindles were measured (eg, single vs double night polysomnography, standard vs hd-EEG); or 4) review articles/conference abstracts; or 5) analysis of the same data set of another manuscript without providing new data on the associations between sleep spindle activity and symptoms; or 6) cognitive functions and positive/negative symptoms not measured using validated measures.

There was no restriction on language or publication dates.

### 2.3 Search strategy

A systematic search of Pubmed, Embase, CINAHL and PsycINFO covering all dates up to the search date in May 2020. The search terms used were: [sleep spindle∗ or sigma power or spindle density or spindle frequency or spindle amplitude or spindle duration or integrated spindle activity or spindle number] AND [psychot∗ or psychos∗ or schiz∗] (see Appendix 1 for the full search strategy). A single researcher screened the titles, keywords and abstracts of each study for eligibility. The full text was obtained if a study was considered potentially eligible or the relevance was unclear. A study was rejected if it explicitly indicated that it studied only non-psychotic patients/healthy individuals. Case studies, opinions, editorials, grey literature and conference abstracts were not included. Full texts of all potentially eligible studies were independently assessed for inclusion by two investigators. Discrepancies were resolved through discussion. Introduction, discussion, and bibliographies from full-text articles were examined for any additional studies. Corresponding authors of the included full-text articles were contacted for any on-going studies or unpublished data.

### 2.4 Data extraction and study quality

Data extraction was done independently by two investigators. Information extracted from each study included the sample size, age, sex, diagnosis, measures used to assess cognitive function, positive and negative symptoms, medication status, technical details of EEG and spindle detection, spindle parameters and information used to measure study quality. Two studies did not report an overall score of positive/negative symptoms [34,35] and one study did not report subscores [36]. Since all included studies only reported some sleep spindle parameters, and not all of them addressed associations between spindle parameters and positive/negative symptoms, all corresponding authors were contacted via email for clarification. Study quality was evaluated using the Appraisal Tool for Cross-sectional Studies (AXIS) [37]. This is an appraisal tool developed for evaluating the risk of bias of cross-sectional studies. AXIS includes 20 items covering justification of sample size, representativeness of the sample, addressing non-responders and non-responder bias, use of validated measures, description of statistical methods, internal consistency of data, declaration of funding sources and conflicts of interest (see Appendix 2 for items of AXIS). Each study was given a score from 0 to 20 with a greater score reflecting a better study quality.

### 2.5 Methods of analysis

Quantitative analysis would be carried out if there were 5 or more studies reporting on the same type of correlation between a sleep spindle parameter and a psychometric measure, or when their scores are interconvertible (eg, 5 studies reporting on the correlations between sleep spindle density and positive symptom score). Nevertheless, due to the heterogeneity of data, a meta-analysis was precluded, while a qualitative analysis was conducted. The review was divided into two parts: 1) the association between sleep spindle and cognitive functions; and 2) the association between sleep spindle and positive and negative symptoms (psychopathology scores).

Each spindle parameter correlating with cognitive functions, positive and negative symptoms was discussed, including spindle density, spindle morphology (duration, amplitude, peak frequency), ISA, spindle number, and sigma power. Unless otherwise specified, they are termed generically as “sleep spindle activity”. A critical analysis of evidence was conducted while taking the quality of study into account.

## 3 Results

### 3.1 Study selection

**Fig. 1:**
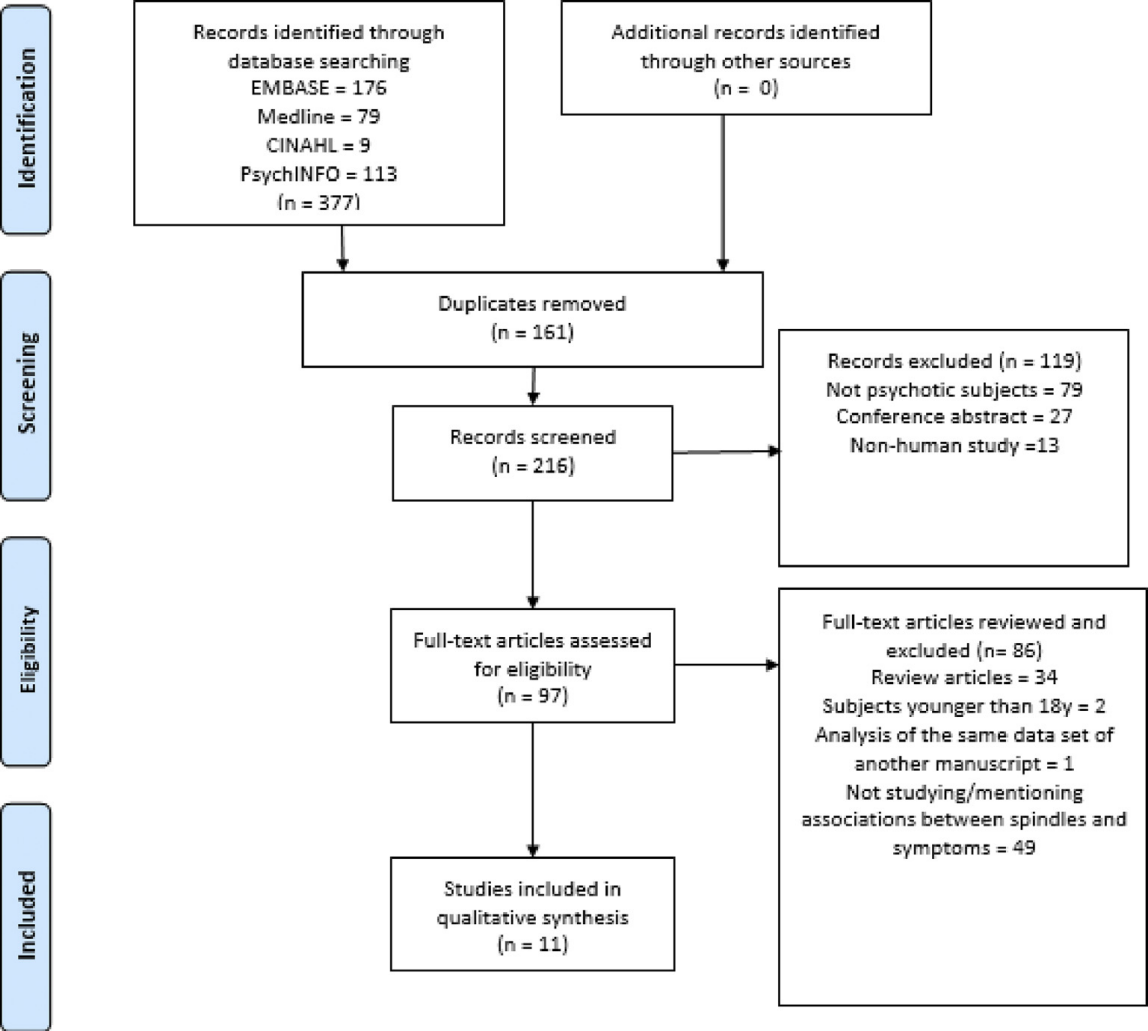
PRISMA flow diagram.

A total of 377 records were identified through electronic database searching (Fig. 1 for the PRISMA flow diagram). After removal of 161 duplicated records, a further 119 records were excluded after title and abstract screening. The full texts of 97 studies were assessed for eligibility. Studies were mostly excluded because they did not analyse the association between sleep spindle activity and symptom severity. In total, 11 studies met the inclusion criteria for this review. Full details for each included study are described in Table 1. Details of excluded studies are described in Appendix 3.

**Table 1:** Subject characteristics and PSG technical details (N = 11).

Study	Subjects	Age (in years)	Sex (M/F)	Illness duration (in years)	Medication status of psychosis/schizophrenia group	Symptom severity	Sleep study technical details
Kaskie et al. [38]	27 FEP	23.2 ± 5.8	17/10	NR	15 antipsychotics naïve 12 on medications	PANSS Positive 20.4 ± 4.6 Negative 16.3 ± 5.0	High density EEG Single night Slow: 12–14 Hz Fast: 14–16 Hz
	23 healthy controls	24.7 ± 5.7	16/7				
Baandrup et al. [35]	37 schizophrenia No control	46.6 ± 9.54	24/13	21.27 ± 11.26	On medications	PANSS Scores NR	Standard PSG Single night C3/C4 Slow: 11–13 Hz Fast: 13–16 Hz
Schilling et al. [39]	17 schizophrenia	29.94 ± 10.60	10/7	5.0 ± 5.4	On medications (Antipsychotics monotherapy)	PANSS Positive 15.1 ± 5.0 Negative 16.1 ± 4.0 General 35.0 ± 8.3	Standard PSG Analysis of second night data F3/F4 Slow: 9–12 Hz Cz Fast: 12–15 Hz
	13 first-degree relatives	33.31 ± 14.05	6/7	–			
	17 healthy controls	26.53 ± 8.78	11/6	–			
Wamsley et al. [40]	21 schizophrenia	34 ± 9	17/4	10 ± 8	On medications	PANSS Positive 12 ± 5 Negative 15 ± 5 General 28 ± 10	Standard PSG Analysis of second night data F3/F4 12–15 Hz
	17 healthy controls	36 ± 7	14/3	–			
Goder et al. [36]	16 schizophrenia	28.3 ± 6.1	7/9	6 ± 4	On medications	PANSS Total: 57 ± 8 (mild) Subscores NR	Standard PSG Analysis of second night data C3/C4 12–16 Hz
	16 healthy controls	29.4 ± 6.4	9/7	–			
Manoach et al. [11]	15 FEP	28 ± 8	11/4	NR	Antipsychotics naïve	FEP: SANS total: 14 ± 3 SAPS total: 9 ± 3 Other psychotic disorders: SANS total: 12 ± 3 SAPS total: 8 ± 5	Standard PSG Analysis of second night data C3/C4 12–15 Hz
	11 Other psychotic disorders	27 ± 7	6/5				
	25 healthy controls	27 ± 7	16/9				
	19 Relatives	14 ± 4	9/10				
Ramakrishnan et al. [41]	20 schizophrenia No control	41.3 ± 8.7	8/12	13.4 ± 8.4	On medications	PANSS Positive 6.5 ± 5.0 Negative 14.5 ± 5.7 General 32.5 ± 8.9	Standard PSG Analysis of second night data C3/C4 Criteria of spindle unclear Only the spindles during the last quarter of sleep were manually evaluated
Keshavan et al. [34]	27 FEP No control	27.2 ± 7.3	18/9	8.4 ± 7.6	Antipsychotics naïve	SAPS, SANS Scores NR	Standard PSG Analysis of second night data C3/C4
Ferrarelli et al. [10]	49 schizophrenia	38.2 ± 10.6	33/16	15 ± 8	On medications	PANSS Positive 21.5 ± 4.2 Negative 22.3 ± 4.8 General 45.2 ± 6.9	High-density EEG Single night Slow: 12–14 Hz Fast: 14–16 Hz
			
	44 healthy controls	36.7 ± 7.8	29/15	–			
Ferrarelli et al. [9]	18 schizophrenia	39.6 ± 9.5	13/5	NR	On medications	PANSS Positive 20.6 ± 3.7 Negative 21.2 ± 3.5 General 42.9 ± 6.2	High-density EEG Single night 12–15 Hz
	17 healthy controls	37.0 ± 10.1					
Forest et al. [42]	8 FEP	31.0 ± 19.9	6/2	NR	Antipsychotics naïve	PANSS Positive 21.0 Negative 23.3 General 85.6 (SD not provided)	Standard PSG Two nights (3 FEP patients could only attend a single night) C3/C4 12–15 Hz
	8 healthy controls	21.4 ± 4.9	6/2				

Abbreviation: EEG, electroencephalography; FEP, first episode psychosis; NR, not reported; PANSS, Positive and Negative Syndrome Scale; PSG, polysomnography; SAPS, Scale for the Assessment of Positive Symptoms; SANS, Scale for the Assessment of Negative Symptoms.

### 3.2 Study characteristics (Table 1)

All 11 studies were published in English, between 2007 and 2020. Eight of them included a control group of healthy individuals. Among these studies, Manoach et al. [11] and Schilling et al. [39] included an additional control group of non-psychotic first-degree relatives. Most studies examined sleep spindles as a whole, but 4 studies separated it into slow and fast spindles [10,35,38,39]. The lower boundaries of slow spindles ranged from 8 to 12 Hz, while the upper boundaries of fast spindles ranged from 15 to 16 Hz. Ferrarelli et al. [43] and Kaskie et al. [38] applied the fixed frequency method to separate spindles at 14 Hz. Schilling et al. Schilling et al. [39] and Baandrup et al. [35] adjusted for individual spindle frequency differences, and spindles were separated at 12 Hz and 13 Hz respectively. There were respectively 8 studies examining the association between sleep spindle activity and cognitive functions, and the relationship between sleep spindle activity and positive/negative symptoms.

Regarding the 8 studies examining cognitive functions, only one study offered a single night hd-EEG [10], while the majority offered double nights standard polysomnography (PSG) (N = 7). Most studies addressed spindle density (N = 5), while other studies addressed spindle morphology (duration, amplitude, peak frequency) (N = 3), sigma power (N = 3), ISA (N = 2) and spindle number (N = 2). A wide range of cognitive tests were employed. Although the majority of studies examined attention (N = 5) and executive function (N = 4), their scores are not interconvertible. Thus, cognitive function tests were considered heterogeneous.

Concerning the 8 studies examining positive and negative symptoms, 3 studies offered a single night hd-EEG [9,10,38], while the rest provided double night standard PSG. Reported sleep spindle parameters were heterogeneous. The most commonly reported parameters were spindle density (N = 5) and spindle morphology (N = 4). Most studies measured positive/negative symptoms with the Positive and Negative Syndrome Scales (PANSS) (N = 6) and the rest applied both the Scale for the Assessment of Positive Symptoms (SAPS) and the Scale for the Assessment of Negative Symptoms (SANS) (N = 2).

### 3.3 Participants characteristics (Table 1)

The sample size of individuals with psychosis in each study was small, ranging from 8 to 49. The total number of individuals with psychotic disorders in studies examining cognitive functions and positive/negative symptoms were 193 and 190, respectively. The age of participants ranged between 18 and 65. Apart from two studies with symptom severity not reported [34,35], subjects in other studies were only mildly severe. Of the studies investigating cognitive functions, three studies recruited drug naïve patients with FEP [11,34,42]. Regarding the studies examining positive and negative symptoms, two studies recruited solely drug-naïve subjects [11,34].

### 3.4 Risk of bias (Appendix 2)

The quality of studies appeared to be low-to-moderate according to the AXIS [37], ranging from 12 to 15 and with only 3 studies scoring 16 out of 20 [35,40,44]. All studies did not justify their sample size and did not address non-responders. 7 studies did not address basic data adequately (eg, illness duration, symptom severity, and PSG technique) [8,11,34,35,38,41,42]. Only one study used random sampling [35], while other studies employed convenient sampling.

### 3.5 Synthesis of results (Table 2)

#### 3.5.1 Primary outcomes

##### 3.5.1.1 Is there a relationship between sleep spindle activity and cognitive function?

6 out of 8 studies examining the relationship between sleep spindle activity and cognitive function reported an increase in sleep spindle activity being associated with improvement in at least one cognitive domain [11,[34], [35], [36],^40^,^42^]. Only 3 of them reported correlation coefficients, with values between 0.46 and 0.75 [36,40,42] and 2 of them used multiple regression analysis [34,35], whereas correlation coefficients of insignificant results were not reported. This association appears to be generalizable across studies on illness course (chronic schizophrenia vs FEP). In summary, a higher sleep spindle activity is associated with better cognitive function. Nevertheless, apart from one study [40], most studies demonstrated that this association does not differentiate between individuals with psychotic disorders and healthy subjects. Thus, this association is a general phenomenon irrespective of illness status.

**Table 2:** Sleep spindle activity and cognitive functions (N = 8).

Study	Cognitive assessment (purpose)	Spindle density	Spindle duration	Spindle amplitude	Peak frequency	Integrated spindle activity	Spindle number	Sigma power
Baandrup et al. [35]	Brief assessment of cognition in schizophrenia (Verbal memory; working memory; motor speed; verbal fluency; attention and processing speed; executive function)	Only motor speed was positively associated with slow sleep spindle density (Beta = 0.70, SE = 0.25, p = 0.008)	No association	No association	No association	NR	NR	NR
Goder et al. [36]	International affective picture system -Learning of pictures before sleep and recognizing ‘new/old’ pictures on the next day (Visual memory)	Sleep spindle density was positively correlated with recognition accuracy for neutral pictures in both patients (r = 0.48, p < 0.05) and healthy controls (r = 0.64, p < 0.01), but no association for emotional pictures.	NR	NR	NR	NR	NR	NR
Manoach et al. [11]	Wisconsin card sort test (Executive function) Trail making tests A and B (Cognitive processing speed, attention, executive function) Block design test (Spatial visualization ability and motor skill) Immediate recall of the California verbal learning test (Verbal memory) Ammons quick test (Verbal IQ) Wide range achievement test-revised, reading portion (Premorbid IQ)	Spindle density was positively associated with better performance on Trails A and B, Wisconsin card sort test, Wide range achievement test-revised, reading portion, and Ammons quick test -All groups (schizophrenia, non-psychotic first-degree relatives and healthy controls) showed similar patterns of association	No association	Spindle amplitude was positively associated with better Trail making test B performance, less WCST perseverative errors, and better Block design test performance (Not different by groups)	No association	NR	NR	No association
Wamsley et al. [40]	Finger tapping motor sequence task (Procedural memory)	Overnight improvement in tapping motor sequence task was positively associated with spindle density (r = 0.45, p = 0.04) No correlation for controls	No association	No association	No association	NR	Overnight improvement in tapping motor sequence task was positively associated with spindle number (r = 0.46, p = 0.04)	No association
Ramakrishnan et al. [41]	Multiple Choice Word Test-B (Premorbid IQ) Digit symbol test (Motor speed, executive function, attention, visuoperceptual function) Controlled oral word association test (Verbal fluency) Tower of London (Executive function) Mirror tracing (Procedural memory)	NR	NR	NR	NR	No relationship between performance and integrated spindle activity. Relationship with IQ not examined.	NR	NR
Keshavan et al. [34]	Ammons quick test (Verbal IQ) Trail making test B (Cognitive processing speed, attention, executive function) Wisconsin card sort test (Executive function)	NR	NR	NR	NR	NR	NR	Spindle power positively correlated with performance on Trail making test B (time and errors) and Wisconsin card sort test (perseverative errors), but not with verbal IQ.
Ferrarelli et al. [10]	Ravens progressive matrices test (Non-verbal IQ)	NR	NR	NR	NR	Ravens progressive matrices test was not associated with integrated spindle activity or spindle number Not reported for controls		NR
Forest et al. [42]	Selective attention task Sustained attention task (Attention)	1. Higher sleep spindle density was associated with lower median reaction times in selective attention task in both patients (r = −0.75, p < 0.05) and controls (r = −0.71, p < 0.05). 2. No correlation for sustained attention task in both patients and controls.	NR	NR	NR	NR	NR	NR

Sleep spindle activity and positive/negative symptoms (N = 8).							
Study	Spindle density	Spindle duration	Spindle amplitude	Frequency	Integrated spindle activity	Spindle number	Sigma power
Kaskie et al. [38]	Negative correlation between negative symptoms and spindle duration (r = −0.60, p = 0.006), and spindle density (r = −0.50, p = 0.025).		No association	NR	NR	NR	NR
Schilling et al. [39]	No association	No association	No association	No association	NR	NR	NR
Goder et al. [36]	No association	NR	NR	NR	NR	NR	NR
Manoach et al. [11]	No association	No association	Positive correlation between spindle amplitude and positive symptoms (No correlation coefficient reported; R^2^ = 0.26, p = 0.05).	No association	NR	NR	NR
Wamsley et al. [40]	No association	No association	Negative correlation between positive symptoms and spindle amplitude (r = −0.47, p = 0.03)	No association	NR	NR	Negative correlation between positive symptoms and sigma power (r = −0.45, p = 0.04).
Keshavan et al. [34]	NR	NR	NR	NR	NR	NR	No association
Ferrarelli et al. [10]	NR	NR	NR	NR	1. Negative correlation between spindle number and stereotyped thinking of PANSS negative symptoms (r = −0.32, p = 0.028). 2. Negative correlation between ISA and spindle number with positive symptoms of PANSS (ISA: −0.40, p = 0.005; spindle number: r = −0.37, p = 0.01). 3. Spindle number was correlated only with conceptual disorganization (r = −0.34, p = 0.03) and hallucinations (r = −0.40, p = 0.01). 4. ISA was correlated exclusively with hallucinations (r = −0.48, p = 0.002).		NR
Ferrarelli et al. [9]	NR	NR	NR	NR	No association	NR	NR

Abbreviation: ISA, Integrated spindle activity; NR, not reported; NREM, non-rapid eye movement; PANSS, Positive and Negative Syndrome Scale.

##### 3.5.1.2 Which cognitive domain is associated with sleep spindle activity?

Cognitive function was evaluated with heterogeneous tests. This review attempted to analyse them as a group, despite knowing that the test results are not interconvertible. The most assessed cognitive domains include: 1) cognitive processing speed and attention; 2) memory; and 3) intelligence.

Cognitive processing speed and attention might have a positive association with sleep spindle activity. It was examined by selective attention tasks [42], trail making tests [11,34] and brief assessment of cognition in schizophrenia [35]. Three out of these four studies [11,34,42] demonstrated a positive relationship. Although inadequate power might be the reason for negative results of the remaining one study [35], its sample size is actually greater than any of the positive studies. In contrast to the negative study which recruited medicated individuals with chronic schizophrenia, participants of positive studies were all FEP who were younger and antipsychotic-naïve.

Three types of memory were assessed: verbal memory [11,35], visual memory [36], and procedural memory [40,41]. While studies consistently showed no relationship between verbal memory and sleep spindle activity, the results were mixed for procedural memory. Again, it is unclear if these results might be due to their underpowered design.

While most studies did not show any relationship between intelligence and sleep spindle activity [10,34,41], Manoach et al. [11] found that both verbal IQ and premorbid IQ to be positively associated with sleep spindle density. The likely reason is difficult to be speculated because these studies applied different spindle parameters and intelligence tests. For instance, only one study measured spindle density [11], while the rest evaluated sigma power and integrated spindle activity.

In summary, while cognitive processing speed and attention might have a positive association with sleep spindle activity in individuals with psychotic disorders, other domains are less conclusive.

##### 3.5.1.3 Is there any relationship between sleep spindle activity and positive/negative symptoms?

The majority of studies did not report any association between sleep spindle activity and positive symptoms [9,34,36,38,39]. For those studies reporting a significant association, the results were mixed. For example, 2 studies reported inverse relationships with positive symptoms (r between −0.34 and −0.48, p < 0.05) [10,40], while 1 study reported a positive association (r not reported) [11].

Associations with negative symptoms is similar to that of positive symptoms, mostly comprised of insignificant results [9,11,34,36,39,40]. Out of the two studies reporting negative correlations with sleep spindle activity (r between −0.32 and −0.60, p < 0.05) [10,38], Ferrarelli et al. [10] only identified a single item of PANSS negative score (stereotyped thinking), but not the whole subscale, having a significant association.

Unfortunately, most studies did not report data on insignificant results rendering quantitative analysis infeasible. Thus, the relationships of sleep spindle activity with both positive and negative symptoms are inconclusive.

#### 3.5.2 Secondary outcomes

##### 3.5.2.1 What is the most consistent corresponding spindle parameter with cognitive function and positive/negative symptoms?

With respect to specific spindle parameters, all studies, which reported on spindle density, consistently reported positive associations between spindle density and cognitive performance [11,35,36,40,42] (see Table 2). Other spindle parameters show an inconsistent relationship with cognitive function. In summary, spindle density appears to be the only spindle parameter having a consistent relationship with cognitive function.

As described above, since the relationship between sleep spindle activity and positive/negative symptoms is inconclusive, any corresponding sleep spindle parameter cannot be identified.

## 4 Discussion

### 4.1 Summary of main results

This systematic review explored the relationship of sleep spindle activity with cognitive and positive/negative symptoms in individuals with psychosis. All cross-sectional studies provided low-to-moderate quality evidence that sleep spindle activity positively correlates with cognitive functions. In contrast, the relationship between spindle activity and positive/negative symptoms is inconclusive. Spindle density appears to be the most consistent parameter which would allow an association with cognitive function to be demonstrated.

### 4.2 Overall completeness and applicability of evidence

The overall completeness is moderate, and the results are applicable to individuals with mild symptoms.

All 8 studies examining the cognitive function had primary aims at investigating the relationship between spindle activity and cognitive functions. Sleep spindle activity positively correlates with cognitive function in both studies involving individuals with chronic schizophrenia and FEP. Furthermore, the studies examined a wide range of cognitive domains. On the other hand, only 5 out of 8 studies examining positive and negative symptoms primarily aimed at investigating the relationship between sleep spindle activity and these symptoms. These studies have good coverage on both individuals with chronic schizophrenia and FEP, as well as different medication status.

Nonetheless, most studies only reported spindle density and morphology. For this reason, it is impossible to draw any conclusion about other less described spindle parameters such as ISA and sigma power. Moreover, most studies only included subjects with mild positive and negative symptoms, which inevitably limits the generalisability of the results. It is understandably difficult to recruit subjects with severe positive symptoms, but it should be feasible to study those with moderate symptoms or predominantly negative symptoms.

### 4.3 Quality of the evidence

The quality of studies as calculated using AXIS is low-to-moderate. Most studies did not state the sample size calculation and recruited only a small sample, resulting in inadequate power. Since most studies used convenient sampling, except one study used random sampling [35], the sampling bias is high. None of the studies address non-responders, or eligible individuals who did not participate. It could be because they had refused to join the study or could not be contacted. They could be from a group with specific features. Therefore, the responding bias is high.

Since studies involved testing of multiple cognitive domains and spindle parameters, multiple comparison should be controlled. Most studies have corrected this problem using statistical non-parametric mapping [9,10,38] and Bonferroni's adjustment [34,35,40]. On the contrary, Manoach et al. [11] failed to address multiple comparison and this might lead to type 1 error.

Regarding key methodological limitations, most studies only reported a correlation coefficient but did not control for confounders like depressive symptoms. The methodology also varied greatly among studies, including the participant profiles, modes of PSG, spindle detection methods and cognitive assessments. However, given the relative paucity of studies, it cannot be decided whether these factors might affect the results.

### 4.4 Potential biases in the review process

A comprehensive search of four search engines was conducted to identify all published studies. Moreover, authors were contacted for any unpublished or newer data since publication. There was also no language restriction. However, there was no attempt to include grey literature since only peer-reviewed academic journals were decided to be included. Since the inclusion of studies and evaluation of study quality were independently undertaken by two investigators, any related biases were minimised.

### 4.5 Agreements and disagreements with other studies or reviews

Other studies and reviews generally agree with our primary outcomes, but they did not address our secondary outcomes.

#### 4.5.1 Primary outcomes

Manoach et al. [31] and Ferrarelli and Tononi [32] conducted narrative reviews summarizing evidence regarding the reduced sleep spindles in schizophrenia. Both authors described that cognitive function has positive association with sleep spindle activity. While they suggest that verbal and procedural memory are associated with sleep spindle activity, our systematic review has found these relationships inconclusive. Our review proposes that the reason for the inconsistent findings would be inadequate sample sizes. On the other hand, we demonstrated that attention and cognitive processing speed might have a positive association with spindle activity in younger drug-naïve individuals, whereas this association is lost in those who were older and medicated. It could be because sleep spindles are lost with aging [45], and associations between spindle activity and cognitive functions diminish. It is difficult to delineate the effect of chronic antipsychotic treatment might have on sleep spindle activity. It is because medicated subjects were treated with different antipsychotics, which can exert differential effect on spindle activity [46,47]. Furthermore, the long-term effect of antipsychotic treatment on sleep spindle activity remains unclear.

As for positive/negative symptoms, these reviews only described positive but not negative symptoms, and they drew conclusions different from the current review. Manoach et al. [31] commented that the relationship between spindle activity and positive symptoms to be inconsistent, which is apparently related to medication status. On the contrary, Ferrarelli and Tononi [32] stated that sleep spindle deficits are associated with worse psychotic symptoms. In the present review, we highlight several studies reporting no association with positive/negative symptoms [9,34,36,39]. Thus, the mixed results do not allow us to conclude a relationship between sleep spindle activity and positive/negative symptoms.

In recent years, spindle-slow oscillation coupling, which is out of the scope of our systematic review, has become a popular area of research. It is believed that accurate temporal coordination between sleep spindles and slow oscillations is vital to memory consolidation [48]. Eszopiclone, a spindle-enhancing agent, failed to improve memory in patients with schizophrenia [49]; B. [50]. This might be because eszopiclone disrupts the timing of spindle-slow oscillation couplings [51]. On the contrary, zolpidem, which similarly acts on GABA_A_ receptors in the thalamus, increases couplings of spindles with slow oscillations [52]. A recent double-blind randomised controlled trial demonstrated that zolpidem enhanced verbal memory in healthy individuals, compared to placebo [53]. Importantly, the enhancement was associated with not only spindle density but also spindle-slow oscillation coupling. Therefore, future studies should investigate slow oscillation along with sleep spindle activity.

#### 4.5.2 Secondary outcomes

Unlike the present systematic review finding spindle density being the best parameter correlating with cognitive function, previous reviews [31,32,54] did not investigate this question. This is important to guide future research to improve comparability.

### 4.6 Conclusions

Based on the low-to-moderate quality of evidence, sleep spindle activity is positively associated with cognitive function, while the relationships with both positive and negative symptoms are inconclusive. Attention and cognitive processing speed appear to have a positive association with sleep spindle activity. Furthermore, spindle density is the most consistent parameter correlating with cognitive function. However, these results might only be applicable to individuals with mild psychotic symptoms.

#### 4.6.1 Implications for practice/research

Due to the limited resources in most centres, the necessity of PSG or hd-EEG in the measurement of sleep spindle activity limits the practicality of our results. We would like to propose some implications for research:

i) In order to improve the quality of future studies, researchers should state the sample size calculation and be adequately sized. Non-responders should also be addressed. All spindle parameters should be reported to facilitate future summarization of evidence.
ii) Although the significance of different spindle detection methods is not apparent in the current review due to paucity of studies, several points should be noted in order to improve methodological uniformity. First, since slow and fast spindles have different topographical distribution [55,56] and are generated at a different times [57], they may have different generating mechanisms and both should, therefore, be analysed. Second, a lower frequency boundary at 9 Hz is suggested as many slow spindles will be missed if the 11 Hz criteria are applied [1]. Finally, detection of spindles should be adjusted for discrete spindle frequency differences as this method is shown to be superior to a fixed frequency method regarding the separation of slow and fast spindles [1,58].
iii) Most studies did not adequately address confounders such as depressive symptoms. Future studies should, therefore, control these confounders to improve spindle detection accuracy.
iv) Since it is unclear whether spindle activity changes with illness course and symptom severity, longitudinal studies are necessary. Medium-to long-term studies of the neuroleptic effect on spindle activity would be valuable.
