# Supplementary material for: Systematic review: the relationship between sleep spindle activity with cognitive functions, positive and negative symptoms in psychosis
Source: Sleep Med X. 2020 Aug 29;2:100025. doi: 10.1016/j.sleepx.2020.100025 (PMC8041130; doi:10.1016/j.sleepx.2020.100025)
Supplement: Multimedia component 1 [file mmc1.pdf]

Appendix 1 Literature search strategy

Database: Embase 1974 to present, Medline 1946 to present, PsycINFO 1806 to present, CINAHL 1961 to present

Search date: 30 May 2020

Search strategy:

1	(sleep adj2 spindle*).ti,ab,kw.
2	(sigma adj2 power).ti,ab,kw.
3	(spindle adj2 density).ti,ab,kw.
4	(spindle adj2 frequency).ti,ab,kw.
5	(spindle adj2 amplitude).ti,ab,kw.
6	(spindle adj2 duration).ti,ab,kw.
7	(integrated adj2 spindle adj2 activity).ti,ab,kw.
8	(spindle adj2 number).ti,ab,kw.
9	1 or 2 or 3 or 4 or 5 or 6 or 7 or 8
10	psychot*.ti,ab,kw.
11	psychos*.ti,ab,kw.
12	schiz*.ti,ab,kw.
13	10 or 11 or 12
14	9 and 13

Appendix 2 Risk of bias table by Appraisal of Cross-sectional Studies (AXIS tool)																					
	1	2	3	4	5	6	7	8	9	10	11	12	13#	14	15	16	17	18	19#	20	Total
Kaskie *et al.* (2019)	Y	Y	N	Y	Y	N	N	Y	Y	Y	Y	N	DK	N	Y	Y	Y	Y	N	Y	14/20
Baandrup *et al.* (2018)	Y	Y	N	Y	Y	Y	N	Y	Y	Y	Y	Y	DK	N	Y	Y	Y	Y	N	Y	16/20
Schilling *et al.* (2017)	Y	Y	N	Y	Y	N	N	Y	Y	Y	Y	Y	DK	N	Y	Y	Y	Y	N	Y	15/20
Goder *et al.* (2015)	Y	Y	N	Y	Y	N	N	Y	Y	Y	Y	Y	DK	N	Y	Y	Y	Y	N	Y	15/20
Manoach *et al.* (2014)	Y	Y	N	Y	Y	N	N	Y	Y	Y	Y	N	DK	N	Y	Y	Y	Y	N	Y	14/20
Wamsley *et al.* (2012)	Y	Y	N	Y	Y	N	N	Y	Y	Y	Y	Y	N	N	Y	Y	Y	Y	N	Y	16/20
Ramakrishnan *et al.* (2012)	Y	Y	N	Y	Y	N	N	Y	Y	Y	Y	N	DK	N	Y	Y	Y	N	N	Y	13/20
Keshavan *et al.* (2011)	Y	Y	N	Y	Y	N	N	Y	Y	Y	Y	N	DK	N	Y	Y	Y	Y	DK	Y	13/20
Ferrarelli *et al.* (2010)	Y	Y	N	Y	Y	N	N	Y	Y	Y	Y	N	DK	N	Y	Y	Y	N	N	Y	13/20
Forest *et al.* (2007)	Y	Y	N	Y	Y	N	N	Y	Y	Y	Y	N	DK	N	Y	Y	Y	N	DK	Y	12/20
Ferrarelli *et al.* (2007)	Y	Y	N	Y	Y	N	N	Y	Y	Y	Y	Y	DK	N	Y	Y	Y	Y	N	Y	15/20

Reverse scoring

Abbreviations: Y, Yes; N, No; DK, Don't know

Appendix 2 Appraisal of Cross-sectional Studies (AXIS tool)

Introduction

1 Were the aims/objectives of the study clear?

Methods

2 Was the study design appropriate for the stated aim(s)?

3 Was the sample size justified?

4 Was the target/reference population clearly defined? (Is it clear who the research was about?)

5 Was the sample frame taken from an appropriate population base so that it closely represented the target/reference population under investigation?

6 Was the selection process likely to select subjects/participants that were representative of the target/reference population under investigation?

7 Were measures undertaken to address and categorise non-responders?

8 Were the risk factor and outcome variables measured appropriate to the aims of the study?

9 Were the risk factor and outcome variables measured correctly using instruments/measurements that had been trialled, piloted or published previously?

10 Is it clear what was used to determined statistical significance and/or precision estimates? (E.g. p-values, confidence intervals)

11 Were the methods (including statistical methods) sufficiently described to enable them to be repeated?

Results

12 Were the basic data adequately described?

13 Does the response rate raise concerns about non-response bias?

14 If appropriate, was information about non-responders described?

15 Were the results internally consistent?

16 Were the results presented for all the analyses described in the methods?

Discussion

17 Were the authors' discussions and conclusions justified by the results?

18 Were the limitations of the study discussed?

Other

19 Were there any funding sources or conflicts of interest that may affect the authors' interpretation of the results?

20 Was ethical approval or consent of participants attained?
