# Supplementary material for: Systematic review: the relationship between sleep spindle activity with cognitive functions, positive and negative symptoms in psychosis
Source: Sleep Med X. 2020 Aug 29;2:100025. doi: 10.1016/j.sleepx.2020.100025 (PMC8041130; doi:10.1016/j.sleepx.2020.100025)
Supplement: Multimedia component 2 [file mmc2.docx]

| **Appendix 3 Excluded studies** | |
| --- | --- |
| **Reason for exclusion: Conference abstract (N=27)** | |
| 1 | Denis *et al.* (2019) |
| 2 | Goder *et al.* (2014) |
| 3 | Baran *et al.* (2018b) |
| 4 | Baran *et al.* (2019b) |
| 5 | Baxter *et al.* (2020) |
| 6 | Demanuele *et al.* (2014) |
| 7 | Demanuele *et al.* (2015) |
| 8 | Denis *et al.* (2018a) |
| 9 | Denis *et al.* (2018b) |
| 10 | Ferrarelli *et al.* (2016) |
| 11 | Coon *et al.* (2018) |
| 12 | Coon *et al.* (2019) |
| 13 | Elmore and Gould (2016) |
| 14 | Jones (2014) |
| 15 | Kotorii and Uchimura (2013) |
| 16 | Lai *et al.* (2020) |
| 17 | Manoach (2019) |
| 18 | Manoach *et al.* (2013) |
| 19 | Mylonas *et al.* (2019a) |
| 20 | Mylonas *et al.* (2017) |
| 21 | Sartory *et al.* (2010) |
| 22 | Winkelbeiner *et al.* (2018) |
| 23 | Zillich *et al.* (2019) |
| 24 | Baran *et al.* (2017b) |
| 25 | Frohlich (2017) |
| 26 | Jindal *et al.* (2009) |
| 27 | Kutty (2017) |
| **Reason for exclusion: Not psychotic subjects (N=79)** | |
| 1 | Mikutta *et al.* (2019) |
| 2 | Agustsson *et al.* (2011) |
| 3 | Baker (2011) |
| 4 | Baker *et al.* (2012) |
| 5 | Bastien *et al.* (2009) |
| 6 | Bat-Pitault *et al.* (2017) |
| 7 | Boly *et al.* (2017) |
| 8 | Bothe *et al.* (2019a) |
| 9 | Bothe *et al.* (2019b) |
| 10 | Cabrera *et al.* (2020) |
| 11 | Campbell and Feinberg (2016) |
| 12 | Castelnovo *et al.* (2020) |
| 13 | Cervena *et al.* (2010) |
| 14 | Chatterjee *et al.* (2014) |
| 15 | Clawson *et al.* (2016) |
| 16 | Comai *et al.* (2013) |
| 17 | D’Agostino *et al.* (2018) |
| 18 | D'Agostino *et al.* (2016) |
| 19 | Dang-Vu *et al.* (2017) |
| 20 | Dornbierer *et al.* (2019) |
| 21 | Driver (1996) |
| 22 | Farmer *et al.* (2018) |
| 23 | Galvan (2019) |
| 24 | Geiser *et al.* (2020) |
| 25 | Godbout *et al.* (2013) |
| 26 | Hahn *et al.* (2019) |
| 27 | Hamann *et al.* (2019) |
| 28 | Hanert *et al.* (2017) |
| 29 | Henry (1950) |
| 30 | Hermann *et al.* (2008) |
| 31 | Holz *et al.* (2012) |
| 32 | Jegou *et al.* (2019) |
| 33 | Kleim *et al.* (2016) |
| 34 | Kuula *et al.* (2017) |
| 35 | Lambert *et al.* (2010) |
| 36 | Lambert *et al.* (2013) |
| 37 | Lennox and Collidge (1949) |
| 38 | Lopez *et al.* (2010) |
| 39 | Lustenberger *et al.* (2016) |
| 40 | Lustenberger *et al.* (2015) |
| 41 | Lustenberger *et al.* (2018) |
| 42 | Maier *et al.* (2019) |
| 43 | Martinez *et al.* (2002) |
| 44 | Mensen *et al.* (2018) |
| 45 | Metcalf (1969) |
| 46 | Mikoteit *et al.* (2012) |
| 47 | Mikoteit *et al.* (2013) |
| 48 | Mikoteit *et al.* (2018) |
| 49 | Mishima (2017) |
| 50 | Page *et al.* (2018) |
| 51 | Paterson *et al.* (2009) |
| 52 | Picard-Deland *et al.* (2018) |
| 53 | Piosczyk *et al.* (2013) |
| 54 | Holz *et al.* (2012) |
| 55 | Pisarenco *et al.* (2014) |
| 56 | Porcacchia *et al.* (2013) |
| 57 | Richard-Malenfant *et al.* (2018) |
| 58 | Rihm *et al.* (2016) |
| 59 | Ritter *et al.* (2018) |
| 60 | Rusterholz *et al.* (2018) |
| 61 | Schabus *et al.* (2017) |
| 62 | van Schalkwijk *et al.* (2019) |
| 63 | Seeck-Hirschner *et al.* (2012) |
| 64 | Sopp *et al.* (2018) |
| 65 | Breslin *et al.* (2014) |
| 66 | Spiegel (1982) |
| 67 | Spiegelhalder *et al.* (2012) |
| 68 | Steullet (2016) |
| 69 | Stokes and Prerau (2017a) |
| 70 | Stokes and Prerau (2017b) |
| 71 | Stokes *et al.* (2018) |
| 72 | Tocci *et al.* (2017) |
| 73 | Thankachan *et al.* (2019) |
| 74 | Uygun *et al.* (2018) |
| 75 | van Schalkwijk *et al.* (2020) |
| 76 | Van Sweden (1983) |
| 77 | Visockis *et al.* (2017) |
| 78 | Wilhelm *et al.* (2017) |
| 79 | Yetkin and Aydogan (2018) |
| **Reason for exclusion: Non-human study (N=13)** | |
| 1 | Aguilar *et al.* (2018) |
| 2 | Ang *et al.* (2018) |
| 3 | Beeske *et al.* (2011) |
| 4 | Blanco-Duque *et al.* (2017) |
| 5 | Ghoshal *et al.* (2020) |
| 6 | Katsuki *et al.* (2017) |
| 7 | Kryzhanovskii and Krupina (1988) |
| 8 | McNally *et al.* (2017) |
| 9 | Steullet *et al.* (2019) |
| 10 | Steullet *et al.* (2018a) |
| 11 | Thankachan *et al.* (2019) |
| 12 | Thankachan *et al.* (2015b) |
| 13 | Uygun *et al.* (2017) |
| **Reason for exclusion: Analysis of the same data set of another manuscript (N=1)** | |
| 1 | Demanuele *et al.* (2017) |
| **Reason for exclusion: Subjects younger than 18y (N=2)** | |
| 1 | Tesler *et al.* (2015) |
| 2 | Gerstenberg *et al.* (2020) |
| **Reason for exclusion: Review articles (N=34)** | |
| 1 | Ferrarelli and Tononi (2017) |
| 2 | Frohlich and Lustenberger (2020) |
| 3 | Gardner *et al.* (2014) |
| 4 | Bian *et al.* (2017) |
| 5 | Castelnovo *et al.* (2018) |
| 6 | Castelnovo *et al.* (2016) |
| 7 | Castelnovo *et al.* (2015) |
| 8 | Chan *et al.* (2017) |
| 9 | Cosgrave *et al.* (2018) |
| 10 | Davies *et al.* (2017) |
| 11 | Feige *et al.* (2013) |
| 12 | Fernandez and Lüthi (2020) |
| 13 | Ferrarelli and Tononi (2016) |
| 14 | Ferrarelli (2015) |
| 15 | Ferrarelli and Tononi (2011) |
| 16 | Goder *et al.* (2016) |
| 17 | Hegerl *et al.* (2009) |
| 18 | Kaskie and Ferrarelli (2020) |
| 19 | Keshavan (2014) |
| 20 | Keshavan (2015) |
| 21 | Lu and Goder (2012) |
| 22 | Manoach (2014) |
| 23 | Manoach *et al.* (2020) |
| 24 | Manoach (2019) |
| 25 | Manoach and Stickgold (2019) |
| 26 | Piantoni *et al.* (2016) |
| 27 | Pinault (2017) |
| 28 | Pocivavsek and Rowland (2018) |
| 29 | Sasidharan (2017) |
| 30 | Wilson and Argyropoulos (2012) |
| 31 | Winsky-Sommerer *et al.* (2019) |
| 32 | Włodarczyk (2018) |
| 33 | Young and Wimmer (2017) |
| 34 | Zhang *et al.* (2019) |
| **Reason for exclusion: Not studying/mentioning associations between spindles and symptoms (N=49)** | |
| 1 | Baran *et al.* (2018a) |
| 2 | Baran *et al.* (2015) |
| 3 | Baran *et al.* (2017a) |
| 4 | Baran *et al.* (2019a) |
| 5 | Göder *et al.* (2008) |
| 6 | Guenole *et al.* (2014) |
| 7 | Kim *et al.* (2015) |
| 8 | Manoach *et al.* (2012) |
| 9 | Poulin *et al.* (2003) |
| 10 | Anderer *et al.* (2005) |
| 11 | Buchmann *et al.* (2014) |
| 12 | Eggert *et al.* (2013) |
| 13 | Ferrarelli (2014) |
| 14 | Ghoshal *et al.* (2019) |
| 15 | Goff (2012) |
| 16 | Guazzelli *et al.* (1985) |
| 17 | Hiatt *et al.* (1985) |
| 18 | Irisawa *et al.* (2006) |
| 19 | Kayabekir *et al.* (2016) |
| 20 | Lechinger *et al.* (2018) |
| 21 | Manoach *et al.* (2010) |
| 22 | McCarley (2014) |
| 23 | McCarley (2016) |
| 24 | McKenna *et al.* (2018) |
| 25 | Mehta *et al.* (2018) |
| 26 | Merikanto (2018) |
| 27 | Miwa *et al.* (2017) |
| 28 | Mylonas *et al.* (2020) |
| 29 | Mylonas *et al.* (2019b) |
| 30 | Pan *et al.* (2016) |
| 31 | Purcell (2017) |
| 32 | Sasidharan *et al.* (2017) |
| 33 | Schilling *et al.* (2018) |
| 34 | Scolnick and Pan (2017) |
| 35 | Steullet *et al.* (2018b) |
| 36 | Tarokh (2019) |
| 37 | Thankachan *et al.* (2015a) |
| 38 | Thankachan *et al.* (2017) |
| 39 | Vukadinovic (2011) |
| 40 | Vukadinovic (2012a) |
| 41 | Vukadinovic (2012b) |
| 42 | Vukadinovic (2015) |
| 43 | Wamsley *et al.* (2013) |
| 44 | Warby *et al.* (2012) |
| 45 | Yazihan *et al.* (2017) |
| 46 | Bartsch *et al.* (2019) |
| 47 | Ferrarelli *et al.* (2007) |
| 48 | Manoach and Stickgold (2015) |
| 49 | Tsekou *et al.* (2015) |

**References of excluded studies:**

Aguilar, D. D., Strecker, R. E., Basheer, R. and McNally, J. M. (2018) 'Aberrant sleep, spindle, and EEG power in mGluR5 knockout mice', *Sleep*, 41 (Supplement 1), p. A12.

Agustsson, E. P., Warby, S., Welinder, P., Carrillo, O., Moore, H. E., Mignot, E. and Perona, P. (2011) 'Reliability of sleep spindle identification by experts, non-experts and automated methods', *Sleep*, 1), p. A112.

Anderer, P., Gruber, G., Parapatics, S., Woertz, M., Miazhynskaia, T., Klösch, G., Saletu, B., Zeitlhofer, J., Barbanoj, M. J. and Danker-Hopfe, H. (2005) 'An E-health solution for automatic sleep classification according to Rechtschaffen and Kales: validation study of the Somnolyzer 24× 7 utilizing the Siesta database', *Neuropsychobiology*, 51(3), pp. 115-133.

Ang, G., McKillop, L. E., Purple, R., Blanco-Duque, C., Peirson, S. N., Foster, R. G., Harrison, P. J., Sprengel, R., Davies, K. E., Oliver, P. L., Bannerman, D. M. and Vyazovskiy, V. V. (2018) 'Absent sleep EEG spindle activity in GluA1 (Gria1) knockout mice: relevance to neuropsychiatric disorders', *Transl Psychiatry Psychiatry*, 8(1), p. 154.

Baker, F. C. (2011) 'Sex differences and menstrual-related effects on sleep', *Sleep and Biological Rhythms*, 9 (4), p. 201.

Baker, F. C., Turlington, S. R. and Colrain, I. (2012) 'Developmental changes in the sleep electroencephalogram of adolescent boys and girls', *Journal of sleep research*, 21(1), pp. 59-67.

Baran, B., Correll, D., Vuper, T. C., Morgan, A., Durrant, S. J., Manoach, D. S. and Stickgold, R. (2018a) 'Spared and impaired sleep-dependent memory consolidation in schizophrenia', *Schizophrenia Research*, 199, pp. 83-89.

Baran, B., Demanuele, C., Correll, D., Vuper, T. C., Seicol, B., Fowler, R. A., Callahan, C., Parr, E., Stickgold, R. and Manoach, D. S. (2015) 'Sleep-dependent memory consolidation in individuals with schizophrenia', *Sleep*, 1), p. A96.

Baran, B., Demanuele, C., Vuper, T. C., Seicol, B., Fowler, R. A., Correll, D., Parr, E., Callahan, C. E., Morgan, A., Stickgold, R. and Manoach, D. S. (2017a) 'The effects of eszopiclone on sleep spindles and memory consolidation in schizophrenia: A double-blind randomized trial', *Sleep*, 40 (Supplement 1), p. A415.

Baran, B., Isik Karahanoglu, F., Demanuele, C., Fowler, R. A., Vuper, T. C., Seicol, B., Correll, D., Callahan, C. E., Parr, E., Stickgold, R. and Manoach, D. S. (2017b) 'Integrity of thalamocortical circuitry is associated with sleep spindle deficits in schizophrenia', *Schizophrenia Bulletin*, 43 (Supplement 1), p. S114.

Baran, B., Karahanoglu, F. I., Demanuele, C., Vangel, M., Stickgold, R., Anticevic, A. and Manoach, D. (2018b) 'Abnormal thalamocortical functional connectivity correlates with sleep spindle deficits in schizophrenia', *Biological Psychiatry*, 83 (9 Supplement 1), pp. S218-S219.

Baran, B., Karahanoglu, F. I., Mylonas, D., Demanuele, C., Vangel, M., Stickgold, R., Anticevic, A. and Manoach, D. S. (2019a) 'Increased Thalamocortical Connectivity in Schizophrenia Correlates With Sleep Spindle Deficits: Evidence for a Common Pathophysiology', *Biological Psychiatry : Cognitive Neuroscience and Neuroimaging*, 4(8), pp. 706-714.

Baran, B., Karahanoglu, F. I., Mylonas, D., Denis, D., Keshavan, M., Stickgold, R. and Manoach, D. S. (2019b) 'Sleep and wake biomarkers of psychotic disorders and their relations with thalamocortical connectivity', *Sleep*, 42 (Supplement 1), p. A27.

Bartsch, U., Simpkin, A. J., Demanuele, C., Wamsley, E., Marston, H. M. and Jones, M. W. (2019) 'Distributed slow-wave dynamics during sleep predict memory consolidation and its impairment in schizophrenia', *NPJ Schizophrenia*, 5(1), p. 18.

Bastien, C. H., St-Jean, G., Turcotte, I., Morin, C. M., Lavallée, M. and Carrier, J. (2009) 'Sleep spindles in chronic psychophysiological insomnia', *Journal of Psychosomatic Research*, 66(1), pp. 59-65.

Bat-Pitault, F., Sesso, G., Deruelle, C., Flori, S., Porcher-Guinet, V., Stagnara, C., Guyon, A., Plancoulaine, S., Adrien, J. and Da Fonseca, D. (2017) 'Altered sleep architecture during the first months of life in infants born to depressed mothers', *Sleep medicine*, 30, pp. 195-203.

Baxter, B., Kwok, K., Talbot, C., Zhu, L., Mylonas, D., Stickgold, R. and Manoach, D. S. (2020) 'Evaluating Closed-Loop Auditory Stimulation During Sleep as an Intervention to Improve Memory Consolidation Deficits in Schizophrenia', *Biological Psychiatry*, 87 (9 Supplement), pp. S283-S284.

Beeske, S., Pichat, P., Francon, D., Hurst, W., Gao, Z., Avenet, P. and Griebel, G. (2011) 'Awakening properties of newly discovered highly selective H3 receptor antagonists in rats', *European Neuropsychopharmacology*, 21, p. S262.

Bian, Y., Liang, W., Yue, W., Han, X., Lin, C., Zhang, J., Wang, Z. and Ma, B. (2017) 'Sleep architecture in drug naive patients with schizophrenia: A meta-analysis', *Chinese Mental Health Journal*, 31(3), pp. 208-2014.

Blanco-Duque, C., Ang, G., Purple, R. J., McKillop, L. E., Yagamata, T., Oliver, P. L., Bannerman, D. M. and Vyazovskiy, V. V. (2017) 'Glua1 knockout mice show reduced EEG sleep spindle activity without presenting long-term memory deficits', *Sleep Medicine*, 40 (Supplement 1), p. e38.

Boly, M., Jones, B., Findlay, G., Plumley, E., Mensen, A., Hermann, B., Tononi, G. and Maganti, R. (2017) 'Altered sleep homeostasis correlates with cognitive impairment in patients with focal epilepsy', *Brain*, 140(4), pp. 1026-1040.

Bothe, K., Hirschauer, F., Wiesinger, H. P., Edfelder, J., Gruber, G., Birklbauer, J. and Hoedlmoser, K. (2019a) 'The impact of sleep on complex gross‐motor adaptation in adolescents', *Journal of sleep research*, 28(4), p. e12797.

Bothe, K., Hirschauer, F., Wiesinger, H. P., Edfelder, J. M., Gruber, G., Hoedlmoser, K. and Birklbauer, J. (2019b) 'Gross motor adaptation benefits from sleep after training', *Journal of Sleep Research*, p. e12961.

Breslin, J., Spanò, G., Bootzin, R., Anand, P., Nadel, L. and Edgin, J. (2014) 'Obstructive sleep apnea syndrome and cognition in Down syndrome', *Developmental Medicine & Child Neurology*, 56(7), pp. 657-664.

Buchmann, A., Dentico, D., Peterson, M. J., Riedner, B. A., Sarasso, S., Massimini, M., Tononi, G. and Ferrarelli, F. (2014) 'Reduced mediodorsal thalamic volume and prefrontal cortical spindle activity in schizophrenia', *Biological Psychiatry*, 75(9), p. 378S.

Cabrera, Y., Holloway, J. and Poe, G. R. (2020) 'Sleep Changes Across the Female Hormonal Cycle Affecting Memory: Implications for Resilient Adaptation to Traumatic Experiences', *Journal of Women's Health*, 29(3), pp. 446-451.

Campbell, I. G. and Feinberg, I. (2016) 'Maturational patterns of sigma frequency power across childhood and adolescence: a longitudinal study', *Sleep*, 39(1), pp. 193-201.

Castelnovo, A., D'Agostino, A., Casetta, C., Sarasso, S. and Ferrarelli, F. (2016) 'Sleep Spindle Deficit in Schizophrenia: Contextualization of Recent Findings', *Current Psychiatry Reports*, 18(8), p. 72.

Castelnovo, A., Ferrarelli, F. and D'Agostino, A. (2015) 'Schizophrenia: from neurophysiological abnormalities to clinical symptoms', *Frontiers in psychology*, 6, p. 478.

Castelnovo, A., Graziano, B., Ferrarelli, F. and D'Agostino, A. (2018) 'Sleep spindles and slow waves in schizophrenia and related disorders: main findings, challenges and future perspectives', *European Journal of Neuroscience*, 48(8), pp. 2738-2758.

Castelnovo, A., Zago, M., Casetta, C., Zangani, C., Donati, F., Canevini, M., Riedner, B. A., Tononi, G., Ferrarelli, F., Sarasso, S. and D'Agostino, A. (2020) 'Slow wave oscillations in Schizophrenia First-Degree Relatives: A confirmatory analysis and feasibility study on slow wave traveling', *Schizophrenia Research*, 24, p. 24.

Cervena, K., Espa, F., Merica, H., Deiber, M. P., Perrig, S. and Ibanez, V. (2010) 'Spectral differences during sleep onset period in sleep onset and sleep maintenance insomnia', *Journal of Sleep Research*, 19, pp. 45-46.

Chan, M. S., Chung, K. F., Yung, K. P. and Yeung, W. F. (2017) 'Sleep in schizophrenia: A systematic review and meta-analysis of polysomnographic findings in case-control studies', *Sleep Medicine Reviews*, 32, pp. 69-84.

Chatterjee, A., Solanki, B., Koul, A., Ghoshal, A., Anand, S., Das, G., Mukherjee, J., Chakraborty, D. P. and Saha, S. P. (2014) 'Study of sleep spindles in pediatric epilepsy patients', *Annals of Indian Academy of Neurology*, 17, pp. S168-S169.

Clawson, B. C., Durkin, J. and Aton, S. J. (2016) 'Form and Function of Sleep Spindles across the Lifespan', *Neural plasticity*, 2016, pp. 6936381-6936381.

Comai, S., Ochoa-Sanchez, R., Rainer, Q. and Gobbi, G. (2013) 'Hypnotic and anxiolytic properties of the selective melatonin MT2 receptor partial agonist UCM765', *Neuropsychopharmacology*, 38, p. S576.

Coon, W. G., Mylonas, D., Baran, B., Demanuele, C., Stickgold, R. and Manoach, D. (2018) 'Sleep spindle coherence and density predict sleep-enhanced learning in schizophrenia', *Sleep*, 41 (Supplement 1), pp. A38-A39.

Coon, W. G., Valderrama, M., Varela, C., Amaya, V., Henao, D., Stickgold, R., Wilson, M. and Manoach, D. (2019) 'Human sleep spindles coupled to hippocampal sharp wave ripples have characteristic EEG features', *Sleep*, 42 (Supplement 1), p. A41.

Cosgrave, J., Wulff, K. and Gehrman, P. (2018) 'Sleep, circadian rhythms, and schizophrenia: where we are and where we need to go', *Current opinion in psychiatry*, 31(3), pp. 176-182.

D'Agostino, A., Castelnovo, A., Cavallotti, S., Canevini, M., Marcatili, M., Ferrarelli, F., Riedner, B., Tononi, G. and Sarasso, S. (2016) 'Sleep spindle deficit in schizophrenia: A high-density EEG study in first-degree relatives', *Journal of Sleep Research*, 25 (Supplement 1), p. 371.

D’Agostino, A., Castelnovo, A., Cavallotti, S., Casetta, C., Marcatili, M., Gambini, O., Canevini, M., Tononi, G., Riedner, B. and Ferrarelli, F. (2018) 'Sleep endophenotypes of schizophrenia: slow waves and sleep spindles in unaffected first-degree relatives', *npj Schizophrenia*, 4(1), pp. 1-8.

Dang-Vu, T. T., Hatch, B., Salimi, A., Mograss, M., Boucetta, S., O'Byrne, J., Brandewinder, M., Berthomier, C. and Gouin, J.-P. (2017) 'Sleep spindles may predict response to cognitive-behavioral therapy for chronic insomnia', *Sleep Medicine*, 39, pp. 54-61.

Davies, G., Haddock, G., Yung, A. R., Mulligan, L. D. and Kyle, S. D. (2017) 'A systematic review of the nature and correlates of sleep disturbance in early psychosis', *Sleep Medicine Reviews*, 31, pp. 25-38.

Demanuele, C., Bartsch, U., Baran, B., Khan, S., Vangel, M. G., Cox, R., Hamalainen, M., Jones, M. W., Stickgold, R. and Manoach, D. S. (2017) 'Coordination of Slow Waves With Sleep Spindles Predicts Sleep-Dependent Memory Consolidation in Schizophrenia', *Sleep*, 40(1), p. 01.

Demanuele, C., Bartsch, U., Wamsley, E. J., Shinn, A. K., Goff, D. C., Jones, M. W., Stickgold, R. and Manoach, D. S. (2014) 'The effects of eszopiclone on slow wave modulation of sleep spindles in schizophrenia', *Biological Psychiatry*, 75(9), p. 58S.

Demanuele, C., Hamalainen, M., Baran, B., Luessi, M., Vuper, T. C., Fowler, R. A., Correll, D., Seicol, B., Callahan, C., Parr, E., Khan, S., Murphy, M. J., Dehghani, N., Stickgold, R. and Manoach, D. S. (2015) 'Characterizing sleep spindle abnormalities in schizophrenia as a novel target for improving cognition', *Sleep*, 1), p. A319.

Denis, D., Larson, O., Sato, E., Kohnke, E., Parr, E., Baran, B., Stewart, K., Cragin, C., Pilo, C., Spitzer, C., Iyer, N. E., King, J., Keshavan, M., Manoach, D. S. and Stickgold, R. (2019) 'Impairment of Sleep-Dependent Memory Consolidation and Sleep Spindles in Early-Course Schizophrenia and First-Degree Relatives', *Biological Psychiatry*, 85 (10 Supplement), pp. S288-S289.

Denis, D., Sato, E., Larson, O., Kohnke, E., Stewart, K., Iyer, N., Parr, E., Keshavan, M., Manoach, D. and Stickgold, R. (2018a) 'Sleep in early course psychosis patients and their first-degree relatives', *Early Intervention in Psychiatry*, 12 (Supplement 1), p. 153.

Denis, D., Sato, E., Larson, O., Kohnke, E. J., Parr, E., King, J., Stewart, K., Baran, B., Keshavan, M., Manoach, D. and Stickgold, R. (2018b) 'Sleep-dependent memory consolidation in early course schizophrenia patients and familial high-risk relatives', *Sleep*, 41 (Supplement 1), pp. A369-A370.

Dornbierer, D. A., Baur, D. M., Stucky, B., Quednow, B. B., Kraemer, T., Seifritz, E., Bosch, O. G. and Landolt, H.-P. (2019) 'Neurophysiological signature of gamma-hydroxybutyrate augmented sleep in male healthy volunteers may reflect biomimetic sleep enhancement: a randomized controlled trial', *Neuropsychopharmacology*, 44(11), pp. 1985-1993.

Driver, H. S. (1996) 'Sleep in women', *Journal of psychosomatic research*, 40(3), pp. 227-230.

Eggert, T., Dorn, H., Sauter, C., Nitsche, M. A., Bajbouj, M. and Danker-Hopfe, H. (2013) 'No effects of slow oscillatory transcranial direct current stimulation (tDCS) on sleep-dependent memory consolidation in healthy elderly subjects', *Brain stimulation*, 6(6), pp. 938-945.

Elmore, Z. C. and Gould, K. L. (2016) 'Board Number: B375 Spindle pole localization of CK1 is necessary for mitotic checkpoint function', *Molecular Biology of the Cell*, 27 (25), p. 4346.

Farmer, C. A., Chilakamarri, P., Thurm, A. E., Swedo, S. E., Holmes, G. L. and Buckley, A. W. (2018) 'Spindle activity in young children with autism, developmental delay, or typical development', *Neurology*, 91(2), pp. e112-e122.

Feige, B., Baglioni, C., Spiegelhalder, K., Hirscher, V., Nissen, C. and Riemann, D. (2013) 'The microstructure of sleep in primary insomnia: an overview and extension', *International Journal of Psychophysiology*, 89(2), pp. 171-180.

Fernandez, L. M. and Lüthi, A. (2020) 'Sleep spindles: mechanisms and functions', *Physiological Reviews*, 100(2), pp. 805-868.

Ferrarelli, F. (2014) 'Anatomical volume of interest analysis and sleep spindle source modeling point to a TRN-MD thalamus-prefrontal cortex circuit deficit in schizophrenia', *Neuropsychopharmacology*, 39, p. S86.

Ferrarelli, F. (2015) 'Sleep in patients with schizophrenia', *Current sleep medicine reports*, 1(2), pp. 150-156.

Ferrarelli, F., Huber, R., Peterson, M. J., Massimini, M., Murphy, M., Riedner, B. A., Watson, A., Bria, P. and Tononi, G. (2007) 'Reduced sleep spindle activity in schizophrenia patients', *American Journal of Psychiatry*, 164(3), pp. 483-92.

Ferrarelli, F., Sarasso, S., Riedner, B., Tononi, G. and Lewis, D. (2016) 'EEG abnormalities during sleep and waking point to thalamo-cortical dysfunction in schizophrenia', *Biological Psychiatry*, 79(9), p. 140S.

Ferrarelli, F. and Tononi, G. (2011) 'The thalamic reticular nucleus and schizophrenia', *Schizophrenia Bulletin*, 37(2), pp. 306-15.

Ferrarelli, F. and Tononi, G. (2016) 'What Are Sleep Spindle Deficits Telling Us About Schizophrenia?', *Biological Psychiatry*, 80(8), pp. 577-8.

Ferrarelli, F. and Tononi, G. (2017) 'Reduced sleep spindle activity point to a TRN-MD thalamus-PFC circuit dysfunction in schizophrenia', *Schizophrenia Research*, 180, pp. 36-43.

Frohlich, F. (2017) 'Rational targeting thalamo-cortical oscillations with noninvasive brain stimulation', *Brain Stimulation*, 10 (2), pp. 356-357.

Frohlich, F. and Lustenberger, C. (2020) 'Neuromodulation of sleep rhythms in schizophrenia: Towards the rational design of non-invasive brain stimulation', *Schizophrenia Research*, 27, p. 27.

Göder, R., Fritzer, G., Gottwald, B., Lippmann, B., Seeck-Hirschner, M., Serafin, I. and Aldenhoff, J. B. (2008) 'Effects of olanzapine on slow wave sleep, sleep spindles and sleep-related memory consolidation in schizophrenia', *Pharmacopsychiatry*, 41(3), pp. 92-9.

Galvan, A. (2019) 'The Need for Sleep in the Adolescent Brain', *Trends in Cognitive Sciences*, 24(1), pp. 79-89.

Gardner, R. J., Kersante, F., Jones, M. W. and Bartsch, U. (2014) 'Neural oscillations during non-rapid eye movement sleep as biomarkers of circuit dysfunction in schizophrenia', *European Journal of Neuroscience*, 39(7), pp. 1091-106.

Geiser, T., Hertenstein, E., Feher, K., Maier, J. G., Schneider, C. L., Zust, M. A., Wunderlin, M., Mikutta, C., Kloppel, S. and Nissen, C. (2020) 'Targeting Arousal and Sleep through Noninvasive Brain Stimulation to Improve Mental Health', *Neuropsychobiology*, pp. 1-9.

Gerstenberg, M., Furrer, M., Tesler, N., Franscini, M., Walitza, S. and Huber, R. (2020) 'Reduced sleep spindle density in adolescent patients with early-onset schizophrenia compared to major depressive disorder and healthy controls', *Schizophrenia Research*, 07, p. 07.

Ghoshal, A., Uygun, D., Yang, L., Lopez-Huerta, V., Garcia, M. A., Fu, Z., McNally, J., Zhang, Q., Mao, X., Nicholson, T., Feng, G., Strecker, R., Purcell, S. and Pan, Q. (2019) '49dysfunction of Cacna1i Impaired Sleep Spindles during Nrem', *European Neuropsychopharmacology*, 29 (Supplement 4), p. S1095.

Ghoshal, A., Uygun, D. S., Yang, L., McNally, J. M., Lopez-Huerta, V. G., Arias-Garcia, M. A., Baez-Nieto, D., Allen, A., Fitzgerald, M., Choi, S., Zhang, Q., Hope, J. M., Yan, K., Mao, X., Nicholson, T. B., Imaizumi, K., Fu, Z., Feng, G., Brown, R. E., Strecker, R. E., Purcell, S. M. and Pan, J. Q. (2020) 'Effects of a patient-derived de novo coding alteration of CACNA1I in mice connect a schizophrenia risk gene with sleep spindle deficits', *Transl Psychiatry Psychiatry*, 10(1), p. 29.

Godbout, R., Lambert, A., Tessier, S., Chevrier and Mottron, L. (2013) 'Sleep characteristics in children with high functioning autism: Questionnaire, sleep diary and laboratory data from a non complaining sample', *Sleep*, 1), p. A318.

Goder, R., Weinhold, S., Graf, A., Ballhausen, F., Prehn-Kristensen, A., Junghanns, K. and Baier, P. C. (2014) 'Sleep spindles as biomarkers of memory dysfunction in schizophrenia', *Journal of Sleep Research*, 23, p. 233.

Goder, R., Weinhold, S. L., Baier, P. C. and Junghanns, K. (2016) 'Disorders of memory formation during sleep in neuropsychiatric disorders. [German]', *Somnologie*, 20(1), pp. 16-21.

Goff, D. (2012) 'Memory consolidation deficits in schizophrenia and the combination of D-cycloserine with cognitive remediation', *Neuropsychopharmacology*, 38, pp. S21-S22.

Guazzelli, M., Maggini, C., Landini, G. and Feinberg, I. (1985) 'Similarity of non-REM abnormalities in schizophrenia and depression', *Archives of general psychiatry*, 42(8), pp. 834-835.

Guenole, F., Chevrier, E., Stip, E. and Godbout, R. (2014) 'A microstructural study of sleep instability in drug-naive patients with schizophrenia and healthy controls: sleep spindles, rapid eye movements, and muscle atonia', *Schizophrenia Research*, 155(1-3), pp. 31-8.

Hahn, M., Joechner, A. K., Roell, J., Schabus, M., Heib, D. P., Gruber, G., Peigneux, P. and Hoedlmoser, K. (2019) 'Developmental changes of sleep spindles and their impact on sleep‐dependent memory consolidation and general cognitive abilities: A longitudinal approach', *Developmental science*, 22(1), p. e12706.

Hamann, C., Rusterholz, T., Studer, M., Kaess, M. and Tarokh, L. (2019) 'Association between depressive symptoms and sleep neurophysiology in early adolescence', *Journal of child psychology and psychiatry*, 60(12), pp. 1334-1342.

Hanert, A., Weber, F. D., Pedersen, A., Born, J. and Bartsch, T. (2017) 'Sleep in Humans Stabilizes Pattern Separation Performance', *The Journal of Neuroscience*, 37(50), p. 12238.

Hegerl, U., Sander, C., Olbrich, S. and Schoenknecht, P. (2009) 'Are psychostimulants a treatment option in mania?', *Pharmacopsychiatry*, 42(5), pp. 169-74.

Henry, C. E. (1950) 'Effect on the electroencephalogram of transorbital lobotomy', *Electroencephalography and clinical neurophysiology*, 2(1-4), pp. 187-192.

Hermann, D. M., Siccoli, M., Brugger, P., Wachter, K., Mathis, J., Achermann, P. and Bassetti, C. L. (2008) 'Evolution of neurological, neuropsychological and sleep-wake disturbances after paramedian thalamic stroke', *Stroke*, 39(1), pp. 62-68.

Hiatt, J. F., Floyd, T. C., Katz, P. H. and Feinberg, I. (1985) 'Further evidence of abnormal non-rapid-eye-movement sleep in schizophrenia', *Archives of General Psychiatry*, 42(8), pp. 797-802.

Holz, J., Piosczyk, H., Feige, B., Spiegelhalder, K., Baglioni, C., Riemann, D. and Nissen, C. (2012) 'EEG sigma and slow‐wave activity during NREM sleep correlate with overnight declarative and procedural memory consolidation', *Journal of sleep research*, 21(6), pp. 612-619.

Irisawa, S., Isotani, T., Yagyu, T., Morita, S., Nishida, K., Yamada, K., Yoshimura, M., Okugawa, G., Nobuhara, K. and Kinoshita, T. (2006) 'Increased omega complexity and decreased microstate duration in nonmedicated schizophrenic patients', *Neuropsychobiology*, 54(2), pp. 134-9.

Jegou, A., Schabus, M., Gosseries, O., Dahmen, B., Albouy, G., Desseilles, M., Sterpenich, V., Phillips, C., Maquet, P. and Grova, C. (2019) 'Cortical reactivations during sleep spindles following declarative learning', *Neuroimage*, 195, pp. 104-112.

Jindal, R. D., Montrose, D. M., Miewald, J. M. and Keshavan, M. S. (2009) 'Sleep correlates of cognition in schizophrenia', *Biological Psychiatry*, 65(8), p. 208S.

Jones, M. (2014) 'Decoding sleep-dependent signatures of thalamic-limbic-cortical dysfunction in neurodevelopmental and genetic models of schizophrenia', *Neuropsychopharmacology*, 39, pp. S87-S88.

Kaskie, R. E. and Ferrarelli, F. (2020) 'Sleep disturbances in schizophrenia: what we know, what still needs to be done', *Current Opinion in Psychology*, 34, pp. 68-71.

Katsuki, F., McNally, J. M., Thankachan, S., McKenna, J. T., Brown, R. E., Strecker, R. E. and McCarley, R. W. (2017) 'Optogenetic manipulation of parvalbumin containing gabaergic neurons in the thalamic reticular nucleus alters declarative and nondeclarative memories in mice', *Sleep*, 40 (Supplement 1), pp. A80-A81.

Kayabekir, M., Ceylan, M., Yalcin, A. and Topalotlu, O. (2016) 'The connection between sleep spindles and seizures in schizencephaly: A case report', *Acta Physiologica*, 218 (Supplement 709), p. 9.

Keshavan, M. (2014) 'Sleep EEG studies in schizophrenia: Methodological considerations', *Schizophrenia Research*, 153, p. S87.

Keshavan, M. (2015) 'Dysplasticity, metaplasticity and schizophrenia: Implications for risk, illness progression, and novel preventive interventions', *Neuropsychopharmacology*, 40, p. S33.

Kim, J., Lee, Y., Oh, S., Choi, J. and Jeong, D. (2015) 'Sleep EEG characteristics of patients with schizophrenia', *Sleep*, 1), p. A330.

Kleim, B., Wysokowsky, J., Schmid, N., Seifritz, E. and Rasch, B. (2016) 'Effects of sleep after experimental trauma on intrusive emotional memories', *Sleep*, 39(12), pp. 2125-2132.

Kotorii, N. and Uchimura, N. (2013) 'Neuropathological hypothesis of sleep dysfunction in Schizophrenia', *Clinical Neurophysiology*, 124 (8), pp. e28-e29.

Kryzhanovskii, G. N. and Krupina, N. A. (1988) 'Neuro-pathophysiologic effects during primary hyperactivation of the bed nucleus of the stria terminalis in the rat brain', *Bulletin of Experimental Biology and Medicine*, 106(7), pp. 10-4.

Kutty, B. M. (2017) 'Transcranial alternating current stimulation (tACS) and sleep: Impact on sleep organization', *Sleep and Vigilance*, 1 (2), p. 129.

Kuula, L., Merikanto, I., Makkonen, T., Halonen, R., Lahti, M., Lahti, J., Heinonen, K., Raikkonen, K. and Pesonen, A. K. (2017) 'Schizotypal traits are associated with sleep spindles and rem in adolescence', *Sleep Medicine*, 40 (Supplement 1), pp. e174-e175.

Lai, M., Kelly, S., Hegde, R., Bannai, D., Lizano, P. and Keshavan, M. (2020) 'Investigating Sleep Spindle Density and Schizophrenia: A Meta-Analysis', *Biological Psychiatry*, 87 (9 Supplement), p. S299.

Lambert, A., Tessier, S., Chevrier, E., Scherzer, P., Mottron, L. and Godbout, R. (2013) 'Sleep in children with high functioning autism: Polysomnography, questionnaires and diaries in a non-complaining sample', *Sleep Medicine*, 14, pp. e137-e138.

Lambert, A., Tessier, S., Mottron, L. and Godbout, R. (2010) 'Sleep architecture in children with autism', *Journal of Sleep Research*, 19, p. 209.

Lechinger, J., Heib, D. P. J., Gruber, W. R., Schabus, M., Weinhold, S. L., Goder, R. and Klimesch, W. (2018) 'Measures of interoception during changes in conscious state from wakefulness to sleep and their potential significance for altered awareness in psychiatric conditions', *Journal of Sleep Research*, 27 (Supplement 1), p. 225.

Lennox, M. A. and Collidge, J. (1949) 'Electroencephalographic changes after prefrontal lobotomy: with particular reference to the effect of lobotomy on sleep spindles', *Archives of Neurology & Psychiatry*, 62(2), pp. 150-161.

Lopez, J., Hoffmann, R. and Armitage, R. (2010) 'Reduced sleep spindle activity in early-onset and elevated risk for depression', *Journal of the American Academy of Child & Adolescent Psychiatry*, 49(9), pp. 934-943.

Lu, W. and Goder, R. (2012) 'Does abnormal non-rapid eye movement sleep impair declarative memory consolidation?: Disturbed thalamic functions in sleep and memory processing', *Sleep Medicine Reviews*, 16(4), pp. 389-94.

Lustenberger, C., Boyle, M. R., Alagapan, S., Mellin, J. M., Vaughn, B. V. and Fröhlich, F. (2016) 'Feedback-controlled transcranial alternating current stimulation reveals a functional role of sleep spindles in motor memory consolidation', *Current Biology*, 26(16), pp. 2127-2136.

Lustenberger, C., O’Gorman, R. L., Pugin, F., Tüshaus, L., Wehrle, F., Achermann, P. and Huber, R. (2015) 'Sleep spindles are related to schizotypal personality traits and thalamic glutamine/glutamate in healthy subjects', *Schizophrenia bulletin*, 41(2), pp. 522-531.

Lustenberger, C., Patel, Y. A., Alagapan, S., Page, J. M., Price, B., Boyle, M. R. and Fröhlich, F. (2018) 'High-density EEG characterization of brain responses to auditory rhythmic stimuli during wakefulness and NREM sleep', *Neuroimage*, 169, pp. 57-68.

Maier, J. G., Kuhn, M., Mainberger, F., Nachtsheim, K., Guo, S., Bucsenez, U., Feige, B., Mikutta, C., Spiegelhalder, K. and Klöppel, S. (2019) 'Sleep orchestrates indices of local plasticity and global network stability in the human cortex', *Sleep*, 42(4), p. zsy263.

Manoach, D. (2014) 'Sleep spindle deficits in schizophrenia: A treatable mechanism of impaired cognition?', *Neuropsychopharmacology*, 39, p. S86.

Manoach, D. (2019) 'Reduced sleep spindles in schizophrenia - a treatable endophenotype that links risk genes to impaired cognition?', *European Neuropsychopharmacology*, 29 (Supplement 1), pp. S15-S16.

Manoach, D., Mylonas, D. and Baxter, B. (2020) 'Targeting sleep oscillations to improve memory in schizophrenia', *Schizophrenia Research*, 31, p. 31.

Manoach, D. and Stickgold, R. (2015) 'Sleep, memory and schizophrenia', *Sleep medicine*, 16(5), p. 553.

Manoach, D. and Stickgold, R. (2019) 'Abnormal Sleep Spindles, Memory Consolidation, and Schizophrenia', *Annual Review of Clinical Psychology*, 15, pp. 451-479.

Manoach, D., Thakkar, K., Stroynowski, E., Ely, A., McKinley, S., Wamsley, E., Djonlagic, I., Vangel, M., Goff, D. and Stickgold, R. (2010) 'Reduced overnight consolidation of procedural learning in chronic medicated schizophrenia is related to specific sleep stages', *Journal of Psychiatric Research*, 44(2), pp. 112-20.

Manoach, D., Wamsley, E., Shinn, A., Tucker, M., Ono, K., McKinley, S., Ely, A., Goff, D. and Stickgold, R. (2013) 'Can we improve cognitive function in schizophrenia by treating abnormal sleep?', *Biological Psychiatry*, 73(9), pp. 21S-22S.

Manoach, D., Wamsley, E. J., Shinn, A. K., Tucker, M. A., Ono, K. E., McKinley, S. K., Ely, A. V., Goff, D. C. and Stickgold, R. (2012) 'The effects of eszopiclone on sleep spindles and memory consolidation in schizophrenia', *Sleep*, 1), p. A339.

Martinez, A., Luna, G., Calvo, J. M., Valdes-Cruz, A., Magdaleno-Madrigal, V., Fernandez-Mas, R., Martinez, D. and Fernandez-Guardiola, A. (2002) 'Electroencephalographic spectral (3D) analysis of the effects of the inhalation of industrial volatile organic compounds on the sleep and the attention in the human. [Spanish]', *Salud Mental*, 25(4), pp. 56-67.

McCarley, R. (2014) 'Optogenetic study of the role of parvalbumincontaining thalamic reticular nucleus neurons in spindle generation: Implications for schizophrenia', *Neuropsychopharmacology*, 39, p. S87.

McCarley, R. (2016) 'Optogenetic and cav3.3 channel manipulation of TRN parvalbumin GABAergic neurons in a mouse model of schizophrenia spindle deficit', *Neuropsychopharmacology*, 41 (Supplement 1), pp. S50-S51.

McKenna, J. T., Yang, C., Katsuki, F., Strecker, R. E., Basheer, R., McNally, J. M. and Brown, R. E. (2018) 'Perineuronal nets in the thalamic reticular nucleus regulate neuronal excitability and gate coupling of sleep spindles to cortical slow waves', *Sleep*, 41 (Supplement 1), p. A29.

McNally, J. M., Shukla, C., Thankachan, S., McKenna, J. T., Yang, C., Brown, R. E., McCarley, R. W. and Basheer, R. (2017) 'Local thalamic reticular nucleus inhibition of t-type calcium channels reduces sleep spindles in mice', *Sleep*, 40 (Supplement 1), p. A48.

Mehta, U. M., Ravishankar, V. and Thirthalli, J. (2018) 'Eszopiclone for persistent negative symptoms in schizophrenia - An unintended N-of-1 study', *Schizophrenia Research*, 193, pp. 438-440.

Mensen, A., Poryazova, R., Huber, R. and Bassetti, C. (2018) 'Individual spindle detection and analysis in high-density recordings across the night and in thalamic stroke', *Scientific reports*, 8(1), pp. 1-11.

Merikanto, I. (2018) 'Genetic risk factors for schizophrenia associate with sleep spindle activity in healthy adolescents', *Journal of Sleep Research*, 27 (Supplement 1), pp. 93-94.

Metcalf, D. R. (1969) 'The effect of extrauterine experience on the ontogenesis of EEG sleep spindles', *Psychosomatic Medicine*, 31(5), pp. 393-399.

Mikoteit, T., Brand, S., Beck, J., Perren, S., von Wyl, A., von Klitzing, K., Holsboer-Trachsler, E. and Hatzinger, M. (2012) 'Visually detected NREM Stage 2 sleep spindles in kindergarten children are associated with stress challenge and coping strategies', *The world journal of biological psychiatry*, 13(4), pp. 259-268.

Mikoteit, T., Brand, S., Beck, J., Perren, S., Von Wyl, A., Von Klitzing, K., Holsboer‐Trachsler, E. and Hatzinger, M. (2013) 'Visually detected NREM Stage 2 sleep spindles in kindergarten children are associated with current and future emotional and behavioural characteristics', *Journal of sleep research*, 22(2), pp. 129-136.

Mikoteit, T., Brand, S., Perren, S., von Wyl, A., von Klitzing, K., Kurath, J., Holsboer-Trachsler, E. and Hatzinger, M. (2018) 'Visually detected non-rapid eye movement stage 2 sleep spindle density at age five years predicted prosocial behavior positively and hyperactivity scores negatively at age nine years', *Sleep Med*, 48, pp. 101-106.

Mikutta, C., Feige, B., Maier, J. G., Hertenstein, E., Holz, J., Riemann, D. and Nissen, C. (2019) 'Phase-amplitude coupling of sleep slow oscillatory and spindle activity correlates with overnight memory consolidation', *Journal of Sleep Research*, 28(6), p. e12835.

Mishima, K. (2017) 'Sleep disturbances as prodromes, risk factors and treatment targets in dementia', *Minerva Psichiatrica*, 58(1), pp. 26-39.

Miwa, H., Basheer, R., McKenna, J. T., McNally, J. M., Strecker, R. E., McCarley, R. W. and Brown, R. E. (2017) 'Roles of GAD67 in the thalamic reticular nucleus for regulating sleep spindle generation', *Sleep*, 40 (Supplement 1), pp. A48-A49.

Mylonas, D., Demanuele, C., Baran, B., Cox, R., Stickgold, R. and Manoach, D. (2020) 'Eszopiclone Disrupts the Thalamocortical Dialogue Necessary for Sleep-Dependent Memory Consolidation in Health and Schizophrenia', *Biological Psychiatry*, 87 (9 Supplement), pp. S170-S171.

Mylonas, D., Demanuele, C., Baran, B., Cox, R., Stickgold, R. and Manoach, D. S. (2019a) 'The effects of eszopiclone on spindles, slow oscillations and their coordination in health and schizophrenia', *Sleep*, 42 (Supplement 1), pp. A367-A368.

Mylonas, D., Demanuele, C., Baran, B., Kohnke, E. J., Tocci, C., Stickgold, R., Hamalainen, M. and Manoach, D. (2017) 'Spindle activity related to motor procedural learning in patients with schizophrenia', *Sleep*, 40 (Supplement 1), pp. A419-A420.

Mylonas, D., Tocci, C., Coon, W. G., Baran, B., Kohnke, E. J., Zhu, L., Vangel, M. G., Stickgold, R. and Manoach, D. S. (2019b) 'Naps reliably estimate nocturnal sleep spindle density in health and schizophrenia', *Journal of Sleep Research*, p. e12968.

Page, J., Lustenberger, C. and Frӧhlich, F. (2018) 'Social, motor, and cognitive development through the lens of sleep network dynamics in infants and toddlers between 12 and 30 months of age', *Sleep*, 41(4), p. zsy024.

Pan, J., Allen, A., Huang, L. and Daez, D. (2016) 'Characterizing rare mis-sense variations of CACNA1I identified in a Swedish schizophrenia cohort', *European Psychiatry*, 33 (SUPPL.), pp. S182-S183.

Paterson, L. M., Nutt, D. J., Durant, C. and Wilson, S. J. (2009) 'Efficacy of Trazodone in primary insomnia; A double-blind randomised placebo controlled polysomnographic study', *European Neuropsychopharmacology*, 3), pp. S385-S386.

Piantoni, G., Halgren, E. and Cash, S. S. (2016) 'The Contribution of Thalamocortical Core and Matrix Pathways to Sleep Spindles', *Neural Plasticity*, 2016, p. 3024342.

Picard-Deland, C., Carr, M., Paquette, T., Saint-Onge, K. and Nielsen, T. (2018) 'Sleep spindle and psychopathology characteristics of frequent nightmare recallers', *Sleep Medicine*, 50, pp. 113-131.

Pinault, D. (2017) 'A neurophysiological perspective on a preventive treatment against schizophrenia using transcranial electric stimulation of the corticothalamic pathway', *Brain Sciences*, 7(4).

Piosczyk, H., Holz, J., Feige, B., Spiegelhalder, K., Weber, F., Landmann, N., Kuhn, M., Frase, L., Riemann, D. and Voderholzer, U. (2013) 'The effect of sleep‐specific brain activity versus reduced stimulus interference on declarative memory consolidation', *Journal of sleep research*, 22(4), pp. 406-413.

Pisarenco, I., Caporro, M., Prosperetti, C. and Manconi, M. (2014) 'High-density electroencephalography as an innovative tool to explore sleep physiology and sleep related disorders', *International Journal of Psychophysiology*, 09, p. 09.

Pocivavsek, A. and Rowland, L. M. (2018) 'Basic Neuroscience Illuminates Causal Relationship Between Sleep and Memory: Translating to Schizophrenia', *Schizophrenia Bulletin*, 44(1), pp. 7-14.

Porcacchia, P., Mora Granizo, F. and Botebol Benhamou, G. (2013) 'Sleep related rhythmic movements and periodic limb movements in schizophrenia: A clinical case', *Sleep Medicine*, 14, p. e234.

Poulin, J., Daoust, A. M., Forest, G., Stip, E. and Godbout, R. (2003) 'Sleep architecture and its clinical correlates in first episode and neuroleptic-naive patients with schizophrenia', *Schizophrenia Research*, 62(1-2), pp. 147-53.

Purcell, S. (2017) 'Sleep spindles and schizophrenia genetics', *Neuropsychopharmacology*, 43 (Supplement 1), pp. S36-S37.

Richard-Malenfant, C., Ray, L., Shlik, J., Downs, N., Gosselin, A., Fogel, S., De Koninck, J. and Robillard, R. (2018) 'Sleep spindles and depressive symptoms in post-traumatic stress disorder', *Journal of Sleep Research*, 27 (Supplement 1), pp. 342-343.

Rihm, J. S., Sollberger, S. B., Soravia, L. M. and Rasch, B. (2016) 'Re-presentation of olfactory exposure therapy success cues during non-rapid eye movement sleep did not increase therapy outcome but increased sleep spindles', *Frontiers in human neuroscience*, 10, p. 340.

Ritter, P., Schwabedal, J., Brandt, M., Schrempf, W., Brezan, F., Krupka, A., Sauer, C., Pfennig, A., Bauer, M. and Soltmann, B. (2018) 'Sleep spindles in bipolar disorder–a comparison to healthy control subjects', *Acta Psychiatrica Scandinavica*, 138(2), pp. 163-172.

Rusterholz, T., Hamann, C., Markovic, A., Schmidt, S. J., Achermann, P. and Tarokh, L. (2018) 'Nature and nurture: brain region-specific inheritance of sleep neurophysiology in adolescence', *Journal of neuroscience*, 38(43), pp. 9275-9285.

Sartory, G., Van Beekum, A., Lohrmann, T. and Pietrowsky, R. (2010) 'Memory consolidation during sleep in schizophrenia', *Schizophrenia Research*, 117 (2-3), p. 480.

Sasidharan, A. (2017) 'Sleep and schizophrenia', *Sleep and Vigilance*, 1 (2), pp. 125-126.

Sasidharan, A., Kumar, S., Nair, A. K., Lukose, A., Marigowda, V., John, J. P. and Kutty, B. M. (2017) 'Further evidences for sleep instability and impaired spindle-delta dynamics in schizophrenia: a whole-night polysomnography study with neuroloop-gain and sleep-cycle analysis', *Sleep Medicine*, 38, pp. 1-13.

Schabus, M., Griessenberger, H., Gnjezda, M.-T., Heib, D. P., Wislowska, M. and Hoedlmoser, K. (2017) 'Better than sham? A double-blind placebo-controlled neurofeedback study in primary insomnia', *Brain*, 140(4), pp. 1041-1052.

Schilling, C., Gappa, L., Schredl, M., Streit, F., Treutlein, J., Frank, J., Deuschle, M., Meyer-Lindenberg, A., Rietschel, M. and Witt, S. H. (2018) 'Fast sleep spindle density is associated with rs4680 (Val108/158Met) genotype of catechol-O-methyltransferase (COMT)', *Sleep*, 41(3), p. 01.

Scolnick, E. and Pan, J. (2017) 'CACNA1I: A potential novel target for schizophrenia motivated by emerging human genetict', *Schizophrenia Bulletin*, 43 (Supplement 1), p. S63.

Seeck-Hirschner, M., Baier, P. C., Weinhold, S. L., Dittmar, M., Heiermann, S., Aldenhoff, J. B. and Göder, R. (2012) 'Declarative memory performance is associated with the number of sleep spindles in elderly women', *The American Journal of Geriatric Psychiatry*, 20(9), pp. 782-788.

Sopp, M. R., Michael, T. and Mecklinger, A. (2018) 'Effects of early morning nap sleep on associative memory for neutral and emotional stimuli', *Brain research*, 1698, pp. 29-42.

Spiegel, R. (1982) 'Aspects of sleep, daytime vigilance, mental performance and psychotropic drug treatment in the elderly', *Gerontology*, 28(Suppl. 1), pp. 68-82.

Spiegelhalder, K., Regen, W., Feige, B., Holz, J., Piosczyk, H., Baglioni, C., Riemann, D. and Nissen, C. (2012) 'Increased EEG sigma and beta power during NREM sleep in primary insomnia', *Biol Psychol*, 91(3), pp. 329-33.

Steullet, P. (2016) 'Oxidative stress affects parvalbumin interneurons structure and function in the thalamic reticular nucleus early during development', *Neuropsychopharmacology*, 41 (Supplement 1), p. S51.

Steullet, P., Bandarabadi, M., Adamantidis, A., Do, K. Q. and Herrera, C. G. (2018a) *JOURNAL OF SLEEP RESEARCH*. WILEY 111 RIVER ST, HOBOKEN 07030-5774, NJ USA.

Steullet, P., Cabungcal, J. H., Bukhari, S., Pantazopoulos, H., Cuenod, M., Berretta, S. and Do, K. Q. (2019) 'S.11.03 Similar anomalies in the thalamic reticular nucleus of a mice model of redox dysregulation and of schizophrenia patients', *European Neuropsychopharmacology*, 29 (Supplement 1), p. S16.

Steullet, P., Cabungcal, J. H., Bukhari, S. A., Ardelt, M. I., Pantazopoulos, H., Hamati, F., Salt, T. E., Cuenod, M., Do, K. Q. and Berretta, S. (2018b) 'The thalamic reticular nucleus in schizophrenia and bipolar disorder: role of parvalbumin-expressing neuron networks and oxidative stress', *Molecular Psychiatry*, 23(10), pp. 2057-2065.

Stokes, P. and Prerau, M. (2017a) 'Seeking a new standard: A novel characterization of sleep spindles through time-frequency peak analysis', *Sleep Medicine*, 40 (Supplement 1), pp. e268-e269.

Stokes, P. and Prerau, M. (2017b) 'Striving for a objective standard: A datadriven approach to spindle detection and characterization', *Sleep*, 40 (Supplement 1), p. A43.

Stokes, P., Rath, P., Manoach, D., Stickgold, R. and Prerau, M. (2018) 'Characterizing clinical population differences in transient oscillation features in the sleep EEG', *Sleep*, 41 (Supplement 1), p. A370.

Tarokh, L. (2019) 'Sleep neurophysiology in childhood onset schizophrenia', *Swiss Archives of Neurology, Psychiatry and Psychotherapy*, 70 (Supplement 8), p. 6S.

Tesler, N., Gerstenberg, M., Franscini, M., Jenni, O. G., Walitza, S. and Huber, R. (2015) 'Reduced sleep spindle density in early onset schizophrenia: a preliminary finding', *Schizophrenia Research*, 166(1-3), pp. 355-7.

Thankachan, S., Katsuki, F., McKenna, J., Yang, C., Shukla, C., Deisseroth, K., Uygun, D., Strecker, R., Brown, R. and McNally, J. (2019) 'Thalamic reticular nucleus parvalbumin neurons regulate sleep spindles and electrophysiological aspects of schizophrenia in mice', *Scientific reports*, 9(1), pp. 1-16.

Thankachan, S., Katsuki, F., McNally, J., McKenna, J., Strecker, R., Brown, R. and McCarley, R. (2015a) 'Translational optogenetic modeling of spindle deficit in schizophrenia', *Neuropsychopharmacology*, 40, pp. S373-S374.

Thankachan, S., McNally, J. M., McKenna, J. T., Strecker, R. E., Brown, R. E. and McCarley, R. W. (2015b) 'Optogenetic investigations in mice to identify the cellular mechanisms of the thalamic reticular nucleus control of spindles: Implications for schizophrenia', *Sleep*, 1), pp. A53-A54.

Thankachan, S., McNally, J. M., McKenna, J. T., Strecker, R. E., Brown, R. E. and McCarley, R. W. (2017) 'Basal forebrain parvalbumin neurons control thalamic reticular neurons: An optogenetic study investigating spindles and nrem sleep regulation', *Sleep*, 40 (Supplement 1), p. A37.

Tocci, C., Kohnke, E., Mylonas, D., Baran, B., Parr, E., Stickgold, R. and Manoach, D. S. (2017) 'Comparison of spindle density and procedural memory reliability in nap and overnight sleep', *Sleep*, 40 (Supplement 1), p. A419.

Tsekou, H., Angelopoulos, E., Paparrigopoulos, T., Golemati, S., Soldatos, C. R., Papadimitriou, G. N. and Ktonas, P. Y. (2015) 'Sleep EEG and spindle characteristics after combination treatment with clozapine in drug-resistant schizophrenia: a pilot study', *Journal of Clinical Neurophysiology*, 32(2), pp. 159-63.

Uygun, D. S., McNally, J. M., Yang, L., Imaizumi, K., Katsuki, F., Brown, R. E., Mao, X., Nicholson, T., Sidor, M., Zhang, Q., Strecker, R. E., McCarley, R. W., Feng, G. and Pan, J. Q. (2017) 'Abnormal sleep spindle rhythmogenesis in mice bearing a schizophrenia associated coding variant in the CACNA1I gene', *Sleep*, 40 (Supplement 1), pp. A6-A7.

Uygun, D. S., Yang, C., Miwa, H., McKenna, J. T., McNally, J. M., Katsuki, F., Strecker, R. E., Brown, R. E. and Basheer, R. (2018) 'Gabaa receptors of the thalamic reticular nucleus regulate sleep spindles: An in vivo investigation by CRISPR - Cas9 genetic abscission', *Sleep*, 41 (Supplement 1), p. A12.

van Schalkwijk, F. J., Hauser, T., Hoedlmoser, K., Ameen, M. S., Wilhelm, F. H., Sauter, C., Klösch, G., Moser, D., Gruber, G. and Anderer, P. (2020) 'Procedural memory consolidation is associated with heart rate variability and sleep spindles', *Journal of Sleep Research*, 29(3), p. e12910.

van Schalkwijk, F. J., Sauter, C., Hoedlmoser, K., Heib, D. P., Klösch, G., Moser, D., Gruber, G., Anderer, P., Zeitlhofer, J. and Schabus, M. (2019) 'The effect of daytime napping and full‐night sleep on the consolidation of declarative and procedural information', *Journal of sleep research*, 28(1), p. e12649.

Van Sweden, B. (1983) 'Tardive EEG-effects following psychosurgical interventions. [German]', *EEG-EMG Zeitschrift fur Elektroenzephalographie Elektromyographie und Verwandte Gebiete*, 14(1), pp. 12-16.

Visockis, V., Pratt, J., Morris, B., Dunlop, J., Brandon, N. and Sakata, S. (2017) 'Site-dependent effects of optogenetic stimulation in thalamic reticular nucleus on cortical states', *Brain and Neuroscience Advances*, 1, p. 211.

Vukadinovic, Z. (2011) 'Sleep abnormalities in schizophrenia may suggest impaired trans-thalamic cortico-cortical communication: towards a dynamic model of the illness', *European Journal of Neuroscience*, 34(7), pp. 1031-9.

Vukadinovic, Z. (2012a) 'Schizophrenia as a disturbance of cortical sensory maps', *Translational Neuroscience*, 3(4), pp. 388-398.

Vukadinovic, Z. (2012b) 'Similarities between cortical "up" states during slow wave sleep and wakefulness: The implications for schizophrenia', *Translational Neuroscience*, 3(1), pp. 51-55.

Vukadinovic, Z. (2015) 'Sleep spindle reductions in schizophrenia and its implications for the development of cortical body map', *Schizophrenia Research*, 168(1-2), pp. 589-90.

Wamsley, E. J., Shinn, A. K., Tucker, M. A., Ono, K. E., McKinley, S. K., Ely, A. V., Goff, D. C., Stickgold, R. and Manoach, D. S. (2013) 'The effects of eszopiclone on sleep spindles and memory consolidation in schizophrenia: a randomized placebo-controlled trial', *Sleep*, 36(9), pp. 1369-76.

Warby, S., Moore, H. E., Carrillo, O., Faraco, J., Lin, L., Peppard, P. E., Young, T. and Mignot, E. (2012) 'Genome wide association study and confounders of sigma power and sleep spindles', *Sleep*, 1), pp. A16-A17.

Wilhelm, I., Groch, S., Preiss, A., Walitza, S. and Huber, R. (2017) 'Widespread reduction in sleep spindle activity in socially anxious children and adolescents', *Journal of Psychiatric Research*, 88, pp. 47-55.

Wilson, S. and Argyropoulos, S. (2012) 'Sleep in schizophrenia: time for closer attention', *British Journal of Psychiatry*, 200(4), pp. 273-4.

Winkelbeiner, S. A., Muller, S., Feher, K., Dierks, T., Koenig, T. and Grieder, M. (2018) 'Sigma as a potential predictor for schizotypal personality', *Clinical EEG and Neuroscience*, 49 (6), pp. NP45-NP46.

Winsky-Sommerer, R., de Oliveira, P., Loomis, S., Wafford, K., Dijk, D.-J. and Gilmour, G. (2019) 'Disturbances of sleep quality, timing and structure and their relationship with other neuropsychiatric symptoms in Alzheimer’s disease and schizophrenia: insights from studies in patient populations and animal models', *Neuroscience & Biobehavioral Reviews*, 97, pp. 112-137.

Włodarczyk, A. (2018) 'Benzodiazepine use in schizophrenia', *Schizophrenia research*, 195, p. 576.

Yazihan, N., Yetkin, S., Kizilay, E. and Akarsu, E. S. (2017) 'Investigation of sleep and cognitive functions on first episode drug-naive non-affective psychotic patients', *Sleep Medicine*, 40 (Supplement 1), p. e354.

Yetkin, O. and Aydogan, D. (2018) 'Effect of CPAP on sleep spindles in patients with OSA', *Respiratory Physiology & Neurobiology*, 247, pp. 71-73.

Young, A. and Wimmer, R. D. (2017) 'Implications for the thalamic reticular nucleus in impaired attention and sleep in schizophrenia', *Schizophrenia Research*, 180, pp. 44-47.

Zhang, Y., Quinones, G. M. and Ferrarelli, F. (2019) 'Sleep spindle and slow wave abnormalities in schizophrenia and other psychotic disorders: Recent findings and future directions', *Schizophrenia Research*, 18, p. 18.

Zillich, L., Streit, F., Schilling, C., Gappa, L., Schredl, M., Frank, J., Deuschle, M., Meyer-Lindenberg, A., Rietschel, M. and Witt, S. H. (2019) 'T86the Polygenic Risk for Schizophrenia Is Associated with Increased Fast Sleep Spindle Density in Healthy Subjects', *European Neuropsychopharmacology*, 29 (Supplement 5), p. S262.
